# B7-H3 regulates KIF15-activated ERK1/2 pathway and contributes to radioresistance in colorectal cancer

**DOI:** 10.1038/s41419-020-03041-4

**Published:** 2020-10-03

**Authors:** Yanchao Ma, Shenghua Zhan, Huimin Lu, Ruoqin Wang, Yunyun Xu, Guangbo Zhang, Lei Cao, Tongguo Shi, Xueguang Zhang, Weichang Chen

**Affiliations:** 1 Department of Gastroenterology, The First Affiliated Hospital of Soochow University, 188 Shizi Road, Suzhou, China; 2 Jiangsu Key Laboratory of Clinical Immunology, Soochow University, 708 Renmin Road, Suzhou, China; 3 Jiangsu Institute of Clinical Immunology, The First Affiliated Hospital of Soochow University, 708 Renmin Road, Suzhou, China; 4 Institute of Paediatric Research, Affiliated Children’s Hospital of Soochow University, 92 Zhongnan Street, Suzhou, China; 5 Jiangsu Key Laboratory of Gastrointestinal tumor Immunology, The First Affiliated Hospital of Soochow University, 708 Renmin Road, Suzhou, China

**Keywords:** Radiotherapy, Colorectal cancer

## Abstract

As an important modality for the local control of colorectal cancer (CRC), radiotherapy or neoadjuvant radiotherapy is widely applied in the clinic, but radioresistance has become a major obstacle for CRC radiotherapy. Here we reported that B7-H3, an important costimulatory molecule, is associated with radioresistance in CRC. The expression of B7-H3 was obviously increased in CRC cells after irradiation. The enhanced expression of B7-H3 promoted, while the knockdown of B7-H3 inhibited, colony formation and cell activity in CRC cells following radiation treatment. B7-H3 overexpression reduced S phase arrest and protected cell apoptosis induced by radiation, whereas B7-H3 knockdown had the opposite effects. In addition, B7-H3 blockade by 3E8, a specific B7-H3 antibody, significantly sensitized CRC cells to irradiation in vivo. Mechanistic analysis revealed that B7-H3 regulated KIF15 via RNA sequencing, which was in dependent of NF-κB pathway. And small interfering RNA (siRNA)-mediated KIF15 silencing or KIF15 blockade by the inhibitor SB743921 abolished the effect of B7-H3 on radioresistance in vitro and in vivo. Similar to B7-H3, we find that the protein expression levels of KIF15, which showed a positive correlation with B7-H3, was abnormal upregulated in cancer tissues than in adjacent normal tissues and associated with TNM stage. Finally, B7-H3/KIF15 enhanced resistance against irradiation in CRC cells via activating ERK1/2 signaling, a key pathway involved in radioresistance in cancer. Our findings reveal an alternative mechanism by which CRC cells can acquire radioresistance via the B7-H3/KIF15/ERK axis.

## Introduction

Colorectal cancer (CRC) is the third most commonly occurring malignancy and accounts for more than 9% of all cancer-related deaths worldwide^1^. Neoadjuvant radiotherapy/chemoradiotherapy (neoRT/CRT) following surgery has been approved by NCCN and is required for comprehensive therapy for locally advanced stage II and III CRC^2^. In addition, preoperative chemoradiotherapy combined with surgery could improve the locoregional control of CRC. However, the treatment effect of both neoRT/CRT and preoperative chemoradiotherapy is still unsatisfactory because of chemoresistance and radioresistance^3^. Importantly, approximately 50% of CRC patients experience recurrence and metastasis after radiotherapy^4^. Therefore, there is an urgent need to understand the mechanisms of radioresistance and identify biomarkers that predict radioresistance in CRC patients.

As a type I transmembrane protein, B7-H3 belongs to the important immune checkpoint B7 ligand family, which provides a costimulatory signal for T-cells in the tumor microenvironment and promotes tumor progression and escape^5–7^. Many studies have suggested that the aberrant expression of B7-H3 exists in different cancer types, including pancreatic carcinoma^8^, esophageal carcinoma^9^, hepatocellular cancer^10^, colorectal cancer^11^, non-small cell lung cancer^12^ and gastric cancer^13^. The frequency of B7-H3-positive circulating epithelial tumor cells (CETCs) is significantly higher in breast cancer patients who receive radiotherapy than in patients who do not receive irradiation, suggesting that the upregulation of B7-H3 expression on CETCs could be a possible mechanism of acquired radioresistance in breast cancer patients^14^. Nevertheless, the functional roles and underlying signaling cascades of B7-H3 associated with radioresistance in CRC have yet to be investigated.

Herein, we first demonstrated that the expression of B7-H3 was obviously increased in CRC cells after irradiation. Further in vitro and in vivo functional analyses showed that B7-H3 enhanced resistance against irradiation in CRC cells by upregulating KIF15 expression via NF-κB, which activated ERK1/2 signaling, a key pathway involved in radioresistance in cancers^15^. B7-H3 blockade by 3E8, a specific B7-H3 antibody^16^, significantly sensitized CRC cells to irradiation in vivo. Moreover, the expression of B7-H3 was positively correlated with KIF15 expression in CRC tissue samples. Overall, our data suggest that B7-H3 contributes to CRC radioresistance and that targeting this molecule may be beneficial for CRC treatment.

## Materials and methods

### Cell lines and cell culture

NCM460, HCT8, HT29, SW480, SW620, HCT116 and RKO CRC cell lines were purchased from the Chinese Academy of Science Cell Bank and cultured in DMEM and RPMI-1640 medium (BioInd, Beit Haemek, Israel) including 10% fetal bovine serum (FBS, BioInd), 100 U/ml penicillin and 100 mg/ml streptomycin (Gibco, Grand Island, USA) in a humidified atmosphere of 5% CO_2_ at 37 °C.

### Immunohistochemistry

Sections from paraffin-embedded tissues were incubated with a goat anti-human B7-H3 antibody (1:200, R&D Systems, #AF1027), a rabbit anti-human KIF15 antibody (1:2000, Proteintech, #55407-1-AP) or a rabbit anti-human Ki67 antibody (1:500, Abcam, ab15580) overnight at 4 °C. This step was followed by staining (45 min at room temperature) with the corresponding HRP-labeled rabbit anti-goat secondary antibody or goat anti-rabbit secondary antibody (Invitrogen). Next, the sections were visualized by staining with 3,3’-diaminobenzidine (Biocare Medical, CA, USA) and counterstaining with hematoxylin (Sigma). The numbers of Ki67-positive cells and total cells were analyzed using a microscope (Leica, Buffalo Grove, USA).

All sections were then reviewed blindly by two experienced pathologists (Dr. Cao and Dr. Zhan). The scoring criteria for B7-H3 and KIF15 immunostaining were based on clinical data and adopted the semiquantitative immunoreactive score (IRS) system^17^. Briefly, category A (intensity of immunostaining) was scored using the following criteria: 0, negative; 1, weak; 2, moderate; and 3, strong. Category B (percentage of immunoreactive cells) was scored using the following criteria: 1 (0–25%); 2 (26–50%); 3 (51–75%); and 4 (76–100%). Final scores were calculated by multiplying the scores of categories A and B in the same section; the scores ranged from 0 to 12.

### Statistical analysis

All statistical analyses were performed using GraphPad 6.0 statistical software packages. Statistically significant differences between groups were determined using Student’s *t*-test. A *P-*value of <0.05 was considered statistically significant in all cases.

### Other methods

Detailed description of other methods used in this study are provided in Supplementary Materials and Methods.

## Results

### B7-H3 enhances the radioresistance of CRC cells in vitro

**Fig. 1:**
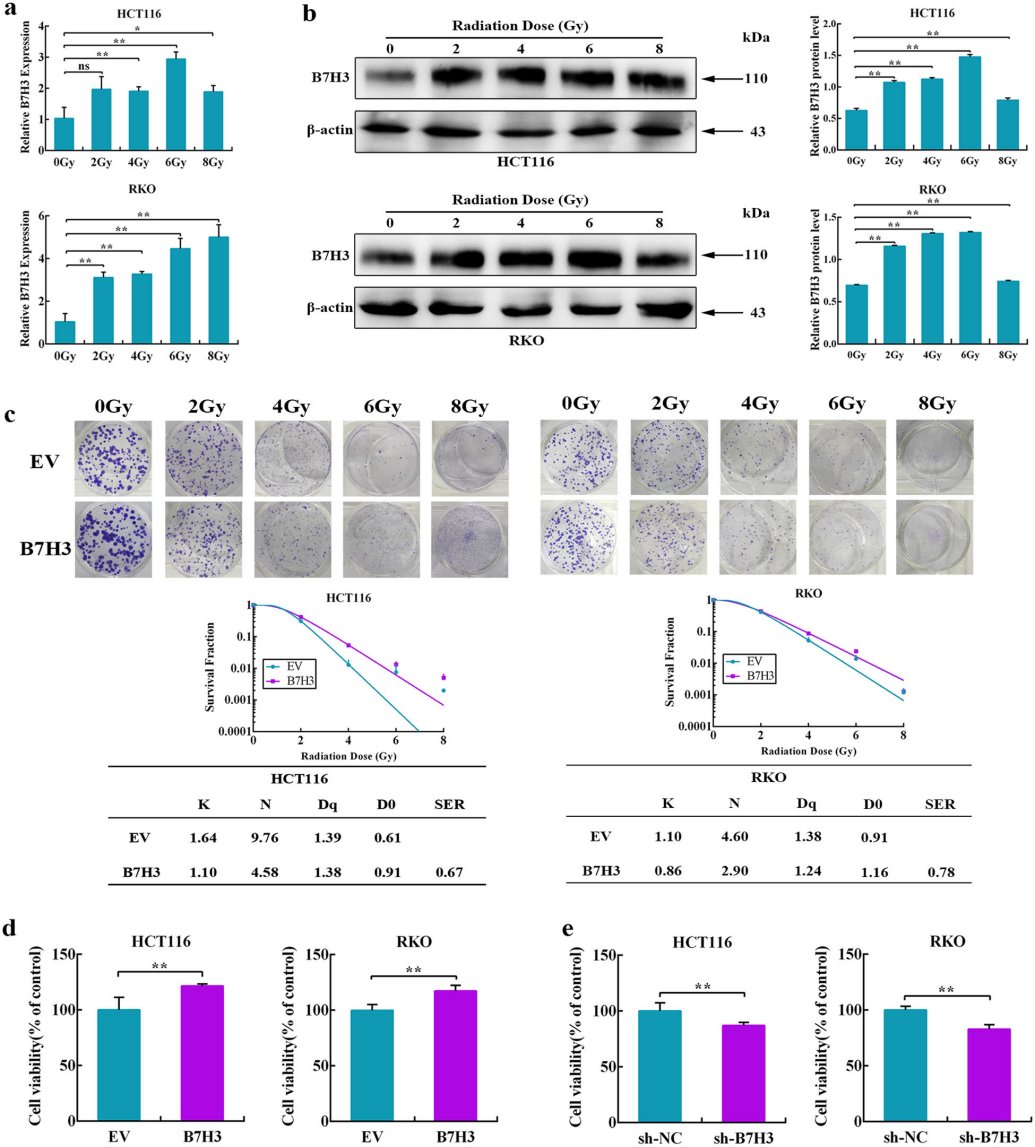
B7-H3 confers to radioresistance of colorectal cells in vitro. **a** RT-qPCR analysis was used to determine the mRNA level of B7-H3 in HCT116 and RKO cells after 0, 2, 4, 6 or 8 Gy X-ray irradiation. **b** Western blot analysis was used to determine the protein level of B7-H3 in HCT116 and RKO CRC cells after 0, 2, 4, 6 or 8 Gy X-ray irradiation. β-actin served as an internal control. Values are expressed as the mean ± SD. **c** Colony formation assay of B7-H3-overexpressing HCT116 or RKO cells exposed to 0, 2, 4, 6 or 8 Gy X-ray irradiation. Up panel: Representative colonies from each group after X-rays irradiation. Down panel: Clonogenic cell survival curves were generated for B7-H3-overexpressing HCT116 and RKO cells after 0, 2, 4, 6 or 8 Gy irradiation. The sensitizer enhancement ratio (SER) was measured using the multitarget, single-hit model. D0, Dq and the calculated SER values of the corresponding groups are shown. **d**, **e** Viability of B7-H3-overexpressing HCT116 and RKO cells **d** or B7-H3-knockdown HCT116 and RKO cells **e** after 4 Gy X-ray irradiation was assessed with CCK8 assays. Values are expressed as the mean ± SD. (*n* = 5). **P < 0.01, *P < 0.05.

As shown in Supplementary Fig. S1a, B7-H3 was frequently upregulated in the CRC cell lines (RKO, HCT116, HCT8, HT29, SW480 and SW620) compared to the human colon healthy cell line (NCM460), suggesting B7-H3 overexpression has a crucial role in CRC progression. To better understand the link between B7-H3 and CRC radioresistance, we first investigated the expression of B7-H3 in CRC cells after ionizing radiation (IR). As shown in Fig. 1a, b, both the mRNA and protein levels of B7-H3 were significantly increased in CRC cells after IR. To determine whether B7-H3 mediated radioresistance in CRC cells, we overexpressed and knocked down B7-H3 in CRC cells. RT-qPCR and Western blot further confirmed that B7-H3 expression was significantly increased in stable B7-H3-overexpressing CRC cell lines (Supplementary Fig. S1b, c), while the B7-H3 level was obviously decreased in stable B7-H3-knockdown CRC cell lines (Supplementary Fig. S1d, e). Next, we noted that B7-H3-overexpressing CRC cells had a significant enhancement in colony formation after IR (SER = 0.67 for B7-H3-overexpressing HCT116 cells and SER = 0.78 for B7-H3-overexpressing RKO cells, Fig. 1c). In contrast, B7-H3-knockdown CRC cells showed significantly fewer colony numbers than their respective scramble control cells after 4 Gy X-ray irradiation (Supplementary Fig. S1f). In addition, cell viability analysis showed that B7-H3 overexpression enhanced the radioresistance of CRC cells (Fig. 1d), while B7-H3 knockdown significantly reduced the viability of CRC cells (Fig. 1e).

**Fig. 2:**
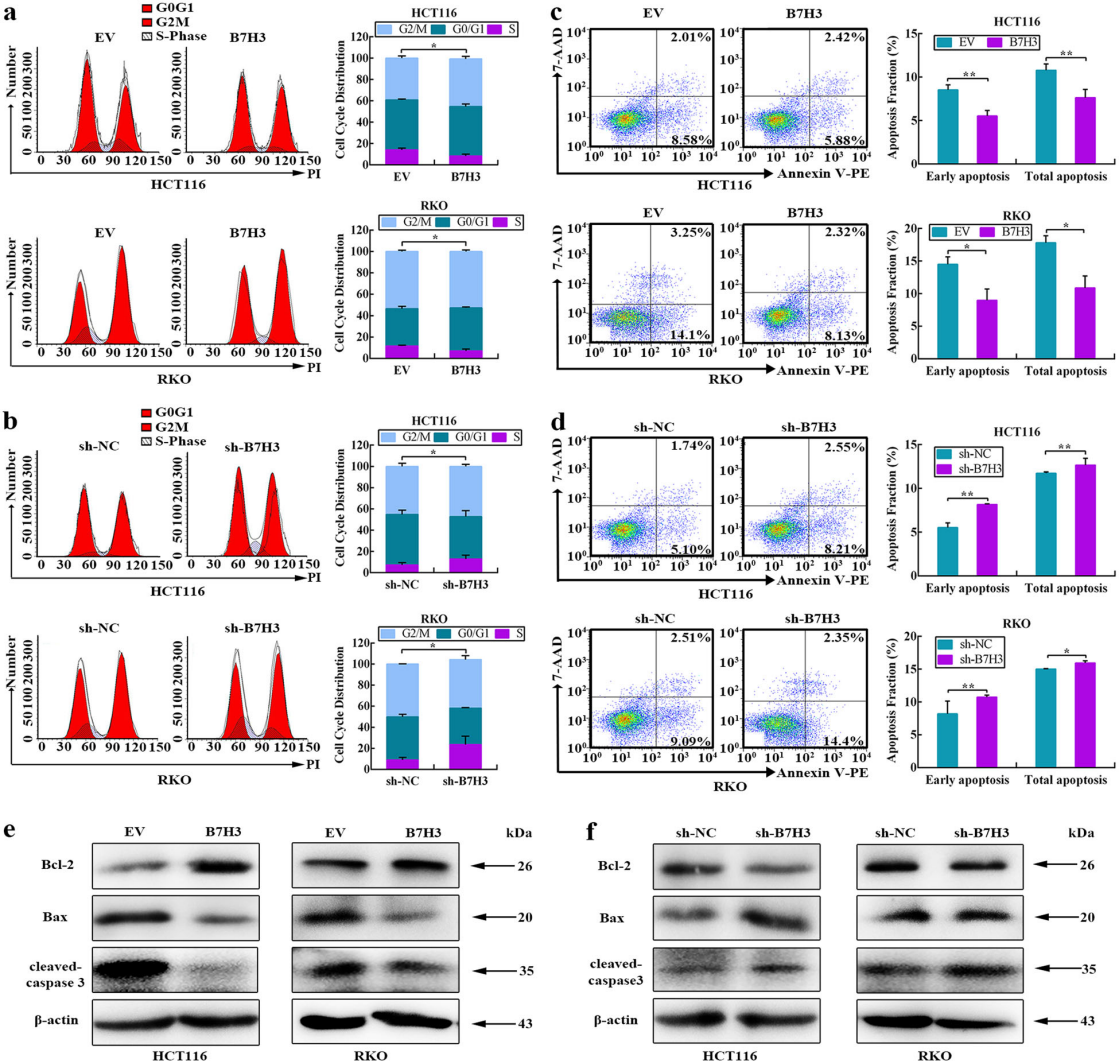
B7-H3 reduces S phase arrest and cell apoptosis of colorectal cells. **a**, **b** The effect of B7-H3 overexpression (**a**) and B7-H3 knockdown (**b**) on the cell cycle distribution in HCT116 and RKO cells after exposure to 4 Gy X-ray irradiation. **c**, **d** Apoptosis was measured using Annexin V/7-AAD double staining in B7-H3-overexpressing (**c**) or B7-H3-knockdown (**d**) CRC cells after exposure to 4 Gy X-ray irradiation. The data are shown as the mean ± SD of three independent experiments. **e**, **f** The protein expression of Bcl-2, Bax and cleaved-caspase 3 in B7-H3-overexpressing (**e**) or B7-H3-knockdown (**f**) CRC cells after exposure to 4 Gy X-ray irradiation. β-actin served as a loading control. **P < 0.01, *P < 0.05.

To investigate whether B7-H3-associated radioresistance was related to cell cycle progression, the cell cycle distribution of B7-H3-overexpressing or B7-H3-knockdown CRC cells after 4 Gy X-ray irradiation was analyzed by flow cytometry. The results showed that the percentage of B7-H3-overexpressing CRC cells in S phase was much lower than that of control cells (Fig. 2a). In contrast, the percentage of B7-H3-knockdown CRC cells in S phase was significantly higher than that of sh-NC cells (Fig. 2b). Moreover, the protein levels of Cyclin B1 and CDK1, two cell cycle-associated genes, were examined in B7-H3-overexpressing or B7-H3-knockdown CRC cells by Western blot. B7-H3-overexpressing reduced, whereas B7-H3 knockdown induced, the expression of CDK1 in CRC cells after 4 Gy X-ray irradiation (Supplementary Fig. S2a, b). However, B7-H3 had no effect on the Cyclin B1 expression (Supplementary Fig. S2a, b).

Next, we used Annexin V/7-AAD double-staining assays to determine the effects of B7-H3 on apoptosis in CRC cells after 4 Gy X-ray irradiation. The results showed that B7-H3 overexpression decreased both the early and total apoptotic populations of CRC cells compared with EV cells after 4 Gy X-ray irradiation (Fig. 2c). By contrast, B7-H3 knockdown increased both the early and total apoptotic populations compared with sh-NC cells (Fig. 2d). Furthermore, enhanced Bcl-2 expression and reduced Bax and cleaved-caspase 3 expression were observed in B7-H3-overexpressing CRC cells after 4 Gy X-ray irradiation (Fig. 2e and Supplementary Fig. S2c), while downregulated Bcl-2 expression and upregulated Bax and cleaved-caspase 3 expression were observed in B7-H3-knockdown CRC cells after 4 Gy X-ray irradiation (Fig. 2f and Supplementary Fig. S2d). These results demonstrate that B7-H3 protects colorectal cancer cells from irradiation.

### B7-H3 blocking antibody 3E8 treatment abrogates B7-H3-mediated CRC cell radioresistance in vivo

Given that B7-H3 exerts a key effect on CRC radioresistance in vitro, we further used 3E8, a special B7-H3 blocking antibody that obviously enhanced the radiosensitivity of CRC cells in vitro (Supplementary Fig. S3a), to further determine the role of B7-H3 in regulating CRC radioresistance. Nude mice bearing empty vector (EV)-HCT116 xenografts were treated with 6 Gy or 10 Gy X-ray. As shown in Supplementary Fig. S3b–d, the tumors of mice in the 10 Gy X-ray irradiation treatment group were smaller than those in the 6 Gy X-ray irradiation treatment group. With reference to previous results^18,19^, we choose 10 Gy for the follow-up experiments in vivo.

**Fig. 3:**
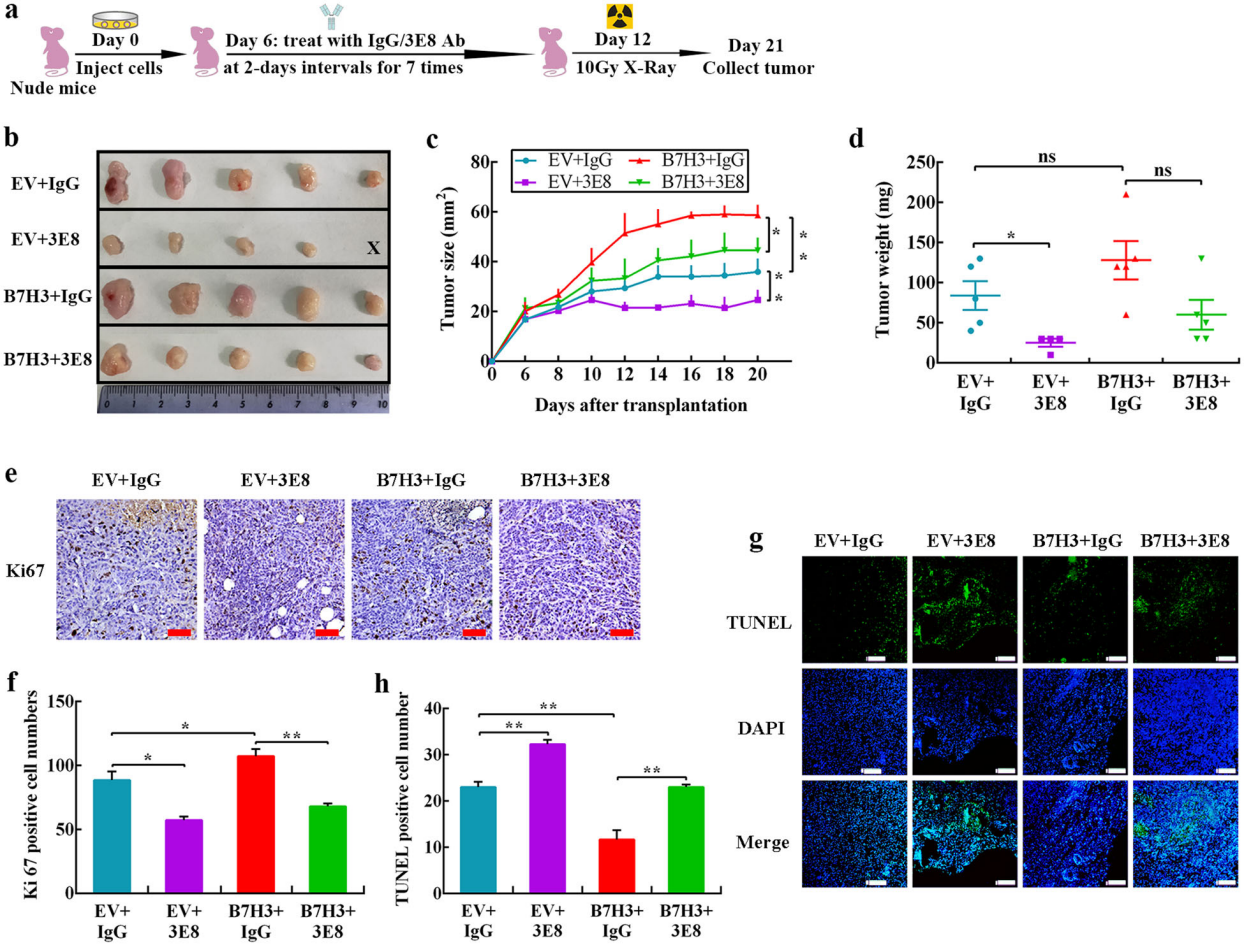
B7-H3 blocking antibody 3E8 treatment abrogates B7-H3-mediated CRC cell radioresistance in vivo. **a** The treatment of each group is shown. HCT116 cells were inoculated under the skin of nude mice. At day 6 after translation, 5 mg/kg 3E8 Ab or IgG was intraperitoneally injected into nude mice 7 times for 2-day intervals. On day 12, the xenograft mice were irradiated locally with 10 Gy X-ray. The tumor size was measured at 2-day intervals. **b** Each group comprised 5 female nude mice. Representative images of tumors formed by EV or B7-H3 cells cotreated with IgG or 3E8 and 10 Gy X-ray irradiation. **c** The growth curves of tumors formed by the indicated EV or B7-H3 cells cotreated with IgG or 3E8 and 10 Gy X-ray irradiation. **d** The weights of tumors formed by the indicated EV or B7-H3 cells cotreated with IgG or 3E8 and 10 Gy X-ray irradiation. The data are presented as the mean ± SEM (*n* = 5 mice per group). **e**, **f** Ki67 IHC staining in tumor tissues of the xenograft model with the indicated treatments (scale bar, 50 μm). **g**, **h** TUNEL staining in tumor tissues of the xenograft model with the indicated treatments (scale bar, 50 μm). The data are presented as the mean ± SEM (*n* = 5 mice per group). **P < 0.01, *P < 0.05.

Nude mice were implanted with EV-HCT116 or B7-H3-HCT116 cells and treated with IgG or 3E8 plus 10 Gy X-ray radiation (Fig. 3a). Compared with the EV + IgG group, the tumors of the B7-H3 + IgG group showed significant resistance to 10 Gy X-ray radiation (Fig. 3b–d). Furthermore, 3E8 treatment obviously suppressed the growth of HCT116 xenografts after 10 Gy X-ray exposure, while 3E8 treatment markedly abrogated the B7-H3-mediated radioresistance of HCT116 xenografts (Fig. 3b–d).

Then, the expression of Ki67, a proliferation marker, was examined by immunohistochemistry (IHC) in xenograft tumor tissues. As shown in Fig. 3e, f, the combined treatment with 3E8 and irradiation significantly reduced the number of Ki67-positive cells in xenografts. The apoptotic cells in the xenograft tissues were also measured with a TUNEL assay. The number of apoptotic cells in the xenograft tissues was significantly increased after the combined treatment with 3E8 and irradiation (Fig. 3g, h). These results together suggest that B7-H3 also contributes to radioresistance of CRC cells in vivo.

### B7-H3 promotes KIF15 expression in CRC after irradiation though NF-κB

**Fig. 4:**
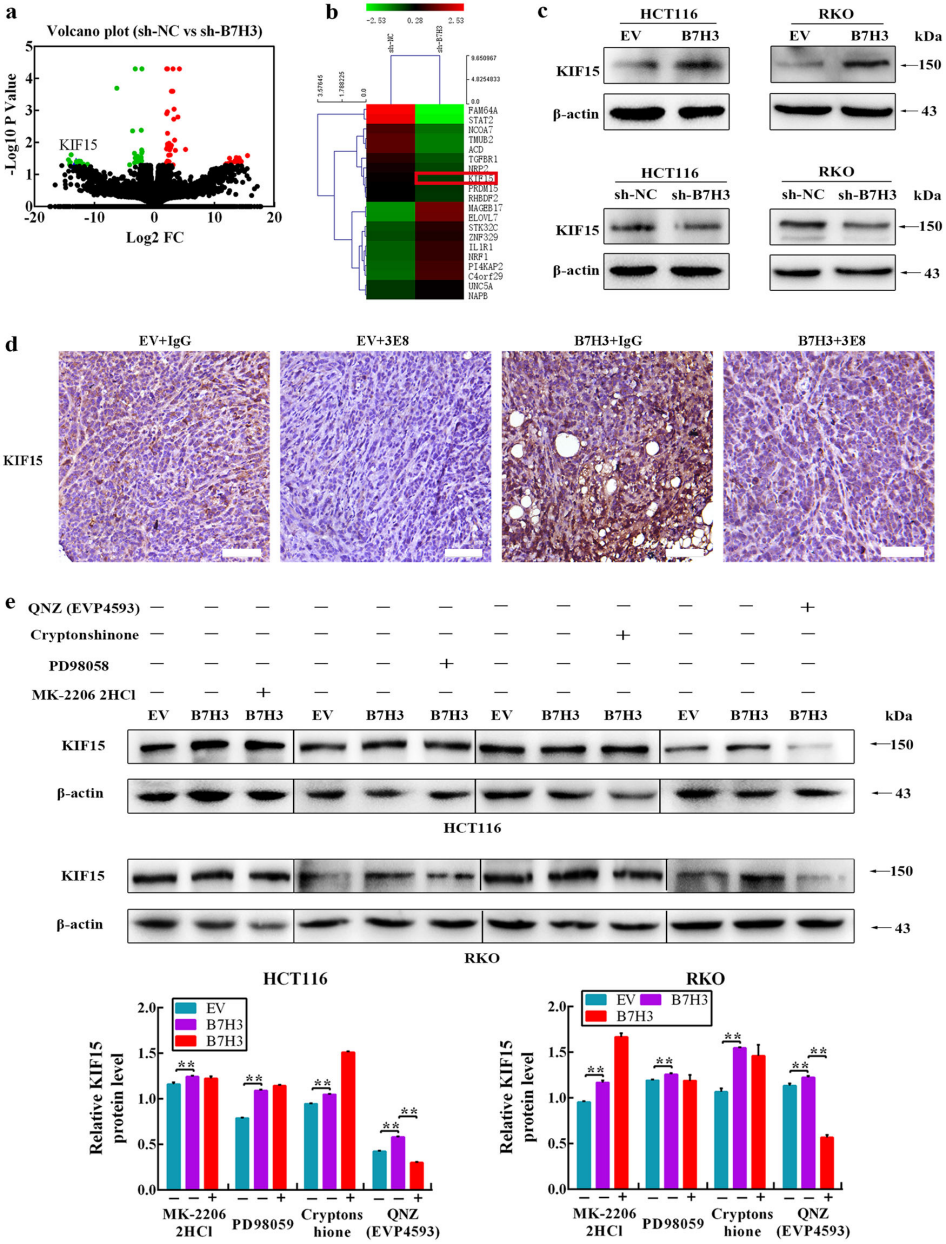
B7-H3 promotes radioresistance via KIF15. **a** Total number of genes with significant changes in gene expression (*P* < 0.05). A total of 208 genes were upregulated (red dots), and 230 genes (green dots) were downregulated. **b** Heatmap of 20 significantly differentially expressed genes (>2-fold) in sh-B7-H3 versus sh-NC RKO cells. Each column represents a cell number. Each row represents a gene. The red color indicates increased expression. The green color indicates decreased expression. **c** The protein expression of KIF15 in B7-H3-overexpressing or B7-H3-knockd own CRC cells after exposure to 4 Gy X-ray irradiation. β-actin served as a loading control. **d** One representative image of the IHC analysis of KIF15 protein expression in 3E8/IgG treatment plus IR xenograft tissue sections (scale bar, 50 µm). **e** The protein expression of KIF15 in B7-H3-overexpressing CRC cells treated with STAT3, AKT, NF-κB, ERK inhibitors prior to treatment with 4 Gy X-ray irradiation. β-actin served as a loading control. Values are expressed as the mean ± SD. **P < 0.01, *P < 0.05.

To decipher the underlying molecular mechanism associated with B7-H3-mediated radioresistance in CRC cells, RNA sequencing (RNA-seq) was performed to profile the transcriptome changes in B7-H3-knockdown RKO cells after 4 Gy X-ray irradiation. In total, 438 genes with significantly differential expression (*P* < 0.05) in B7-H3-knockdown RKO cells were identified. Among these, 208 genes were upregulated, and 230 genes were downregulated (Fig. 4a). A total of 97 genes had a fold change >2, and 38 genes had >2-fold downregulation (Supplementary Table S1). The top 10 upregulated and downregulated genes are summarized in Fig. 4b. We next validated the results of the RNA-seq analysis with RT-qPCR. Consistent with the RNA-seq results, several genes deregulated in the RNA-seq data, including IL1R1, NRF1, STK32C, ZNF329 and UNC5A, were remarkably higher in the sh-B7-H3 group than in the sh-NC group, while the expression of FAM64A, NCOA7, TMUB2, KIF15 and PRDM15 was lower in the sh-B7-H3 group (Supplementary Fig. S4a). We observed that KIF15, a gene encoding a member of the kinesin family of proteins, plays a vital role in regulating the cell cycle^20^. The protein level of KIF15 was upregulated in both B7-H3-overexpressing CRC cells, while the protein level of KIF15 was downregulated in B7-H3-knockdown CRC cells (Fig. 4c and Supplementary Fig. S4b). In 3E8/IgG Ab plus IR treatment xenograft, KIF15 was analyzed by IHC staining. Consistent with the in vitro results, B7-H3 promoted KIF15 expression, while 3E8 treatment reduced the expression of KIF15 (Fig. 4d). These data suggest that B7-H3 could promote KIF15 expression in CRC after IR treatment.

Researches have reported that multiple signaling pathways such as MAPK, NF-κB and STAT3 were downstream targets of B7-H3 in tumors^11,21,22^. Our previous results have shown that the phosphorylation levels of AKT, NF-κB and STAT3 obviously reduced in CRC cells after B7-H3 knockdown^16^. Moreover, abnormal activation of these signal pathways was involved in radioresistance of tumor^23^. Therefore, we hypothesized that B7-H3 may regulate the expression of KIF15 by affecting one or more signaling pathways. To further investigated our surmise, an STAT3 inhibitor (Cryptonshinone, 4 μM), AKT inhibitor (MK-2206 2HCl, 4 μM), ERK inhibitor (PD98059, 2 μM) and NF-κB inhibitor (QNZ EVP4593, 2 μM) were used prior to 4 Gy X-ray irradiation. Western blot assays found that B7-H3 regulates the expression level of KIF15 through the NF-κB signaling pathway after IR (Fig. 4e).

### The B7-H3/KIF15 axis confers radioresistance in vitro and in vivo

**Fig. 5:**
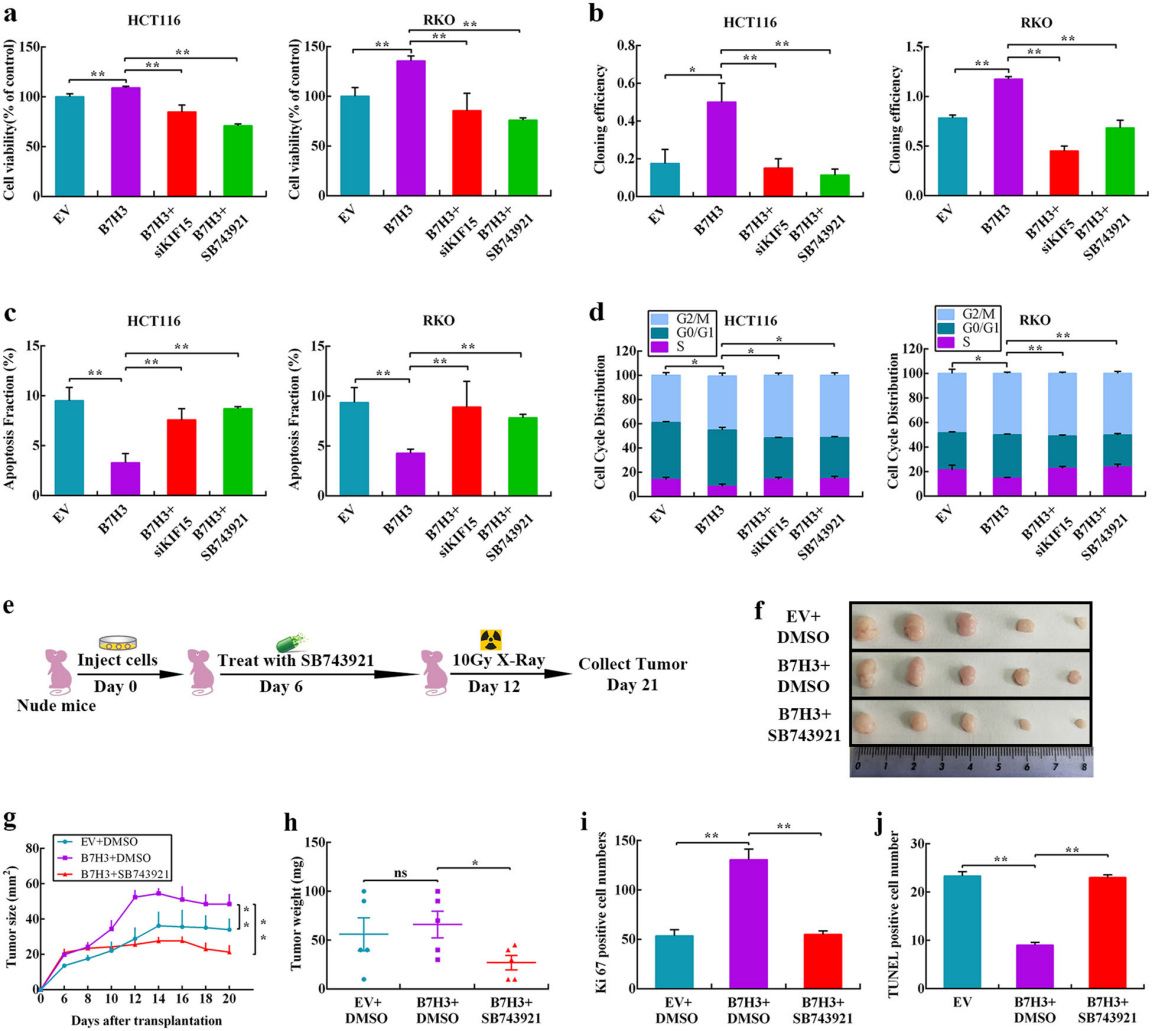
The B7-H3/KIF15 axis confers radioresistance in vitro and in vivo. **a** Viability was assessed in B7-H3-overexpressing HCT116 and RKO cells treated with KIF15 siRNA or SB743921 prior to treatment with 4 Gy X-ray irradiation. **b** Colony formation was assessed in B7-H3-overexpressing HCT116 and RKO cells treated with KIF15 siRNA or SB743921 prior to treatment with 4 Gy X-ray irradiation. **c** Apoptosis was measured using Annexin V/7-AAD double staining in B7-H3-CRC cells treated with KIF15 siRNA or SB743921 prior to treatment with 4 Gy X-ray irradiation. The data are shown as the mean ± SD of three independent experiments. **d** Cell cycle progression was measured in B7-H3-CRC cells treated with KIF15 siRNA or SB743921 prior to treatment with 4 Gy X-ray irradiation. Values are expressed as the mean ± SD. **e** The treatment of each group is shown. HCT116 cells were inoculated under the skin of nude mice. At Day 6 after translation, 2.5 mg/kg SB743921 or DMSO was intraperitoneally injected into nude mice. On Day 12, the xenograft mice were irradiated locally with 10 Gy X-ray. Tumor size was measured at 2-day intervals. **f** Each group was composed of 5 female nude mice. Representative images of tumors formed by EV + DMSO or B7-H3 + DMSO cells with or without SB743921 treatment. **g** The growth curves of tumors formed by the indicated EV + DMSO or B7-H3 + DMSO cells with or without SB743921 treatment. The data are presented as the mean ± SEM (*n* = 5 mice per group). **h** The weights of tumors formed by the indicated EV + DMSO or B7-H3 + DMSO cells with or without SB743921 treatment. The data are presented as the mean ± SEM (*n* = 5 mice per group). **i** Ki67-positive cell numbers in tumor tissues of the nude mouse xenograft model with the indicated treatments. **j** TUNEL-positive cell numbers in tumor tissues of the nude mouse xenograft model with the indicated treatments. The data are presented as the mean ± SEM (*n* = 5 mice per group). **P < 0.01, *P < 0.05.

We hypothesized that B7-H3 may contribute to CRC cell radioresistance by controlling KIF15. To test this hypothesis, an siRNA targeting KIF15, which reduced the expression of KIF15 in CRC cells (Supplementary Fig. S4c, d), or SB743921, a KIF15 inhibitor, was used to treat B7-H3-overexpressing CRC cells. The results of cell viability and colony formation assays showed that both KIF15 knockdown and SB743921 treatment abolished the B7-H3-induced increase in radioresistance (Fig. 5a, b and Supplementary Fig. S4e). Additionally, KIF15 knockdown and SB743921 treatment obviously upregulated the apoptotic population of B7-H3-overexpressing CRC cells after 4 Gy X-ray irradiation (Fig. 5c and Supplementary Fig. S4f). Moreover, KIF15 knockdown reduced the protein level of Bcl-2, while increased the level of Bax and cleaved-caspase 3 in B7-H3-overexpressing CRC cells after 4 Gy X-ray irradiation (Supplementary Fig. S5a). Furthermore, silencing KIF15 and treatment with SB743921 significantly increased the percentage of B7-H3-overexpressing CRC cells in S phase after 4 Gy X-ray irradiation (Fig. 5d and Supplementary Fig. S5b). Additionally, KIF15 knockdown increased the protein level of CDK1 in B7-H3-overexpressing CRC cells after 4 Gy X-ray irradiation (Supplementary Fig. S5c). In summary, these findings reveal that the B7-H3/KIF15 axis contributes to the radioresistance of CRC.

To translate our findings, we assessed whether the B7-H3/KIF15 axis conferred resistance to irradiation in vivo. To address whether inhibiting KIF15 activity could reverse B7-H3-mediated CRC cell radioresistance in vivo, we performed a subcutaneous xenograft tumor assay using B7-H3-HCT116 and respective control cells in athymic nude mice. SB743921 (2.5 mg/kg) in DMSO was intraperitoneally injected into nude mice on day 6, and the tumor radioresponse was detected (Fig. 5e). As shown in Fig. 5f–h, although the tumor weight was not significantly different between the EV + DMSO and B7-H3 + DMSO groups, subcutaneous xenografts in the B7-H3 + DMSO group grew significantly faster than those in the EV + DMSO group following 10 Gy X-ray irradiation, whereas cotreatment with SB743921 and irradiation further suppressed tumor growth and tumor weight. Moreover, the expression of KIF15 was increased in B7-H3 + DMSO than EV + DMSO groups, whereas obviously reduced after cotreatment with SB743921 and irradiation (Supplementary Fig. S5d), in accordance with the in vitro data described above. As shown in Fig. 5i and Supplementary Fig. S5d, B7-H3 significantly induced the number of Ki67-positive cells in the xenografts after 10 Gy X-ray irradiation, while SB743921 treatment significantly reduced the number of Ki67-positive cells. As shown in Fig. 5j and Supplementary Fig. S5e, the number of apoptotic cells in the B7-H3 + DMSO group was significantly decreased relative to that in the EV + DMSO group, whereas SB743921 dramatically increased the number of apoptotic cells in xenografts after 10 Gy X-ray irradiation. These data indicate that B7-H3 promotes radioresistance in vitro and in vivo via KIF15.

### The ERK signaling pathway is required for B7-H3/KIF15 axis-mediated radioresistance in CRC cells

**Fig. 6:**
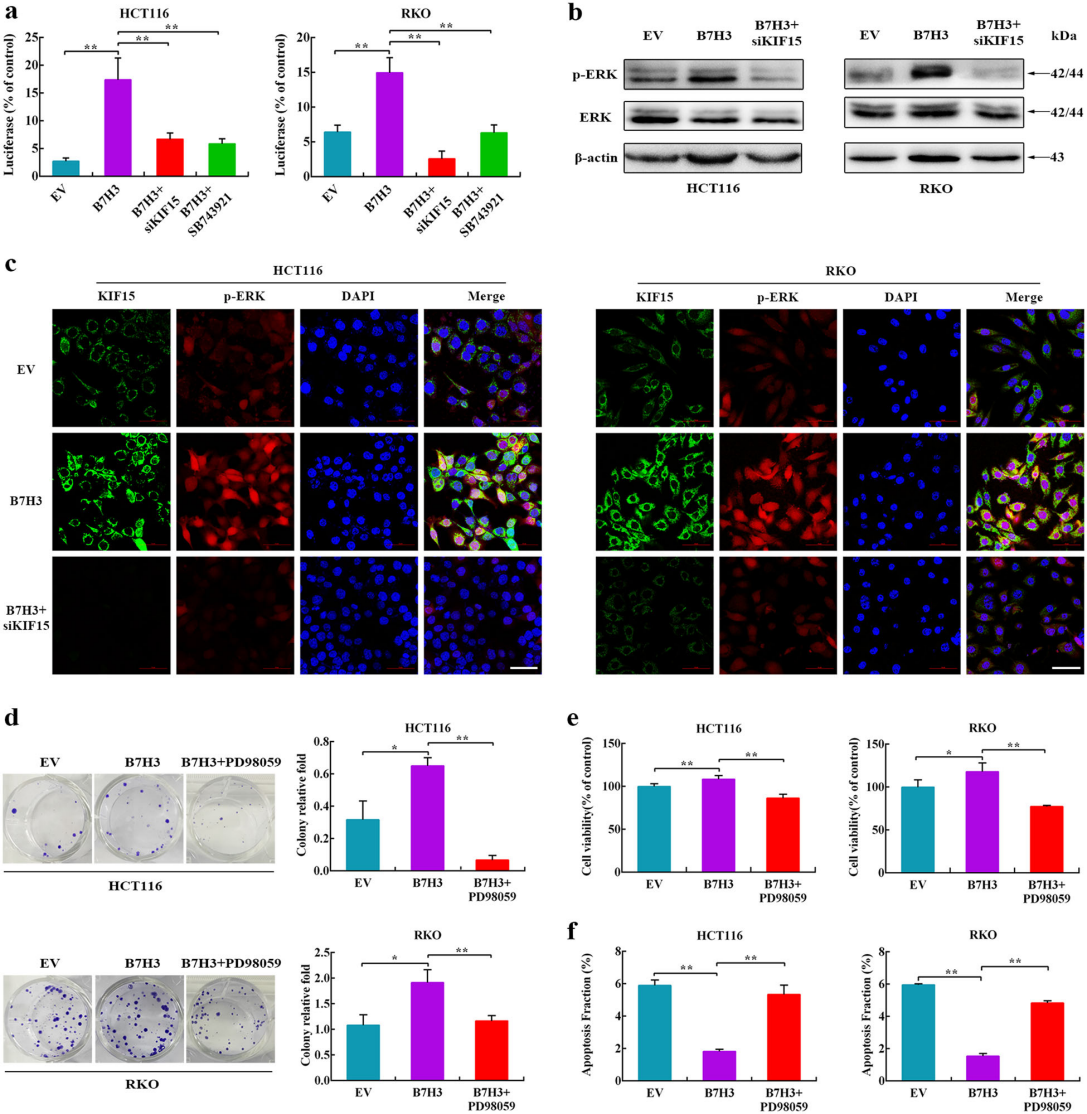
The B7-H3/KIF15-mediated ERK signaling pathway promotes radioresistance in CRC. **a** ERK-responsive luciferase activity was detected in B7-H3-overexpressing CRC cells treated with KIF15 siRNA or SB743921 prior to treatment with 4 Gy X-ray irradiation. **b** ERK activation (examined by the p-ERK expression level) was detected by Western blot in B7-H3-overexpressing HCT116 and RKO cells treated with KIF15 siRNA prior to treatment with 4 Gy X-ray irradiation. β-actin served as a loading control. **c** Immunofluorescence staining was performed to investigate KIF15 and p-ERK expression in B7-H3-overexpressing HCT116 and RKO cells treated with KIF15 siRNA prior to treatment with 4 Gy X-ray irradiation (green, KIF15; red, p-ERK; blue, DAPI nuclear staining, scale bar, 50 µm). **d** Colony formation was assessed in B7-H3-overexpressing HCT116 and RKO cells treated with PD98059 prior to treatment with 4 Gy X-ray irradiation. **e** Viability was assessed in B7-H3-overexpressing HCT116 and RKO cells treated with PD98059 prior to treatment with 4 Gy X-ray irradiation. **f** Apoptosis was measured using Annexin V/7-AAD double staining in B7-H3-overexpressing CRC cells treated with PD98059 prior to treatment with 4 Gy X-ray irradiation. The data are shown as the mean ± SD of three independent experiments. **P < 0.01, *P < 0.05.

A previous study indicated that KIF15 promotes pancreatic cancer proliferation via the MEK-ERK signaling pathway^24^. More importantly, the ERK signaling pathway plays pivotal roles in radioresistance in many human tumors^15^. Thus, we postulated that the B7-H3/KIF15 axis contributes to radioresistance in CRC by activating the ERK signaling pathway. To test this hypothesis, we first detected whether the activity of ERK (determined by the ERK DNA-binding activity) was enhanced by the B7-H3/KIF15 axis using luciferase reporter assays. The results showed that B7-H3 overexpression significantly increased ERK activity in HCT116 and RKO cells after IR. In contrast, silencing KIF15 and treatment with SB743921 significantly decreased ERK activity in B7-H3-overexpressing CRC cells after 4 Gy X-ray irradiation (Fig. 6a). Meanwhile, the phosphorylation level of ERK in CRC cells after 4 Gy X-ray irradiation was determined by Western blot analysis. As shown in Fig. 6b, B7-H3 overexpression significantly enhanced the ERK phosphorylation level in CRC cells after 4 Gy X-ray irradiation. Conversely, KIF15 knockdown reversed the upregulation of the ERK phosphorylation level in B7-H3-overexpressing CRC cells (Fig. 6b and Supplementary Fig. S6a). We further performed immunofluorescence (IF) assay to illustrate that B7-H3/KIF15 axis could upregulate ERK phosphorylation in CRC cells. The expression plasmids carrying human B7-H3 cDNA significantly increased the protein levels of B7-H3 in HCT116 and RKO cells (Supplementary Fig. S6b). The results of IF assay showed that B7-H3 overexpression significantly enhanced the fluorescence intensity of both KIF15 and phosphorylated ERK, wheras KIF15 knockdown counteracted this effect (Fig. 6c). Collectively, these results suggest that the B7-H3/KIF15 signaling cascade is involved in the enhancement of radioresistance via the ERK pathway in CRC cells.

To assess whether ERK signaling is key for B7-H3/KIF15 axis-mediated radioresistance in CRC cells, colony formation, cell viability, cell apoptosis and Western blot assays were performed in B7-H3-overexpressing CRC cells treated with PD98059, an ERK inhibitor, plus 4 Gy X-ray radiation. As shown in Fig. 6d–f and Supplementary Fig. S6c-d, treatment with PD98059 abolished B7-H3-induced radioresistance in CRC cells.

### Correlation analysis between B7-H3 and KIF15 in CRC tissue samples

**Fig. 7:**
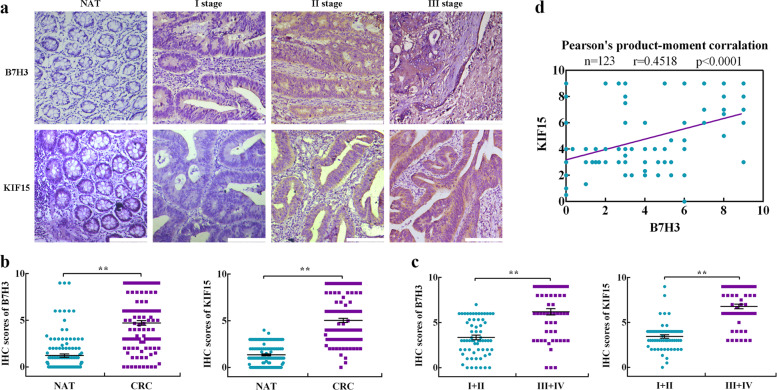
Correlation analysis between B7-H3 and KIF15 in CRC tissue samples. **a** One representative image of the IHC analysis of B7-H3 and KIF15 protein expression in CRC (*n* = 123) tissue sections at different stages (scale bar, 50 µm). **b** B7-H3 and KIF15 protein expression based on the staining index in nonmalignant adjacent tissues (NATs) and CRC specimens. **c** B7-H3 and KIF15 protein expression based on the staining index in CRC specimens at different clinical stages. Values are expressed as the mean ± SEM. **d** Correlation analysis of the staining index of the expression levels of B7-H3 and KIF15 proteins in human CRC specimens (*n* = 123). **P < 0.01, *P < 0.05.

Our previous studies have shown B7-H3 is aberrantly expressed in CRC and contributes to drug resistance^7,22^. To further investigate the correlations between B7-H3 and KIF15 protein levels, we analyzed 123 cancer tissues and adjacent normal tissues of patients with CRC by IHC staining. The results showed that the protein expression levels of both B7-H3 and KIF15 were much higher in cancer tissues than in adjacent normal tissues (Fig. 7a, b). Additionally, it was observed that B7-H3 and KIF15 expression increased with tumor stage. The levels of B7-H3 and KIF15 were higher in advanced clinical stages (III and IV) than in early stages (I and II) (Fig. 7a, c and Supplementary Table S2). Pearson’s analysis showed a positive correlation between the levels of B7-H3 and KIF15 in CRC patients (Fig. 7d). These results indicate that B7-H3 and KIF15 are positively correlated in human CRC specimens.

## Discussion

Radiotherapy is a common treatment modality for solid tumors, including CRC^3^. However, radioresistance, resulting in tumor recurrence and a poor prognosis, is still a major reason for treatment failure^2^. Previous studies have indicated that B7-H3 regulates various tumor biological processes, including radioresistance^14,25^. Consistent with these findings, we found that the B7-H3 mRNA and protein expression levels were significantly increased in CRC cells after X-ray irradiation. Moreover, B7-H3 overexpression promoted, while B7-H3 knockdown inhibited, the radioresistance of CRC in vitro and in vivo. Hence, we conclude that B7-H3 may play a critical role in the regulation of radioresistance in CRC cells.

Mounting evidence indicates that immune checkpoint blockade combined with radiotherapy can acquire considerable success in multiple tumor types^26–28^. Anti-PD-L1 antibody treatment can improve the response of pancreatic ductal adenocarcinoma to radiotherapy and enhance the effect of radiotherapy in preventing the formation of liver metastases^27^. Given the key immunologic and nonimmunologic functions of B7-H3 in tumors^7,10,14,25^, it will be valuable to develop blocking antibodies against B7-H3 for tumor therapy. 8H9, which targets B7-H3, combined with craniospinal irradiation has been determined to prolong the survival of patients with metastatic central nervous system neuroblastoma^29^. Moreover, enoblituzumab (MGA271), which directly and specifically targets B7-H3, has also been applied to Phase I trials for different pediatric tumors^30^. In this study, we found that 3E8, a specific B7-H3 blocking antibody, significantly sensitized CRC cells to irradiation in the xenografts of nude mice. Future prospective trials to investigate the effects of 3E8 on the antitumor immune responses of tumors with or without radiotherapy are warranted.

As a critical member of the KIF family, prior studies have noted the importance of KIF15 in the progression of various cancers^20,24,31^. KIF15 is differentially expressed in breast cancer and adjacent tissues, and high levels of KIF15 are significantly correlated with the poor overall survival of patients with breast cancer^32^. KIF15 knockdown induces G1/S phase cell cycle arrest and inhibits cell growth in human lung adenocarcinoma cell lines^20^. In the present study, we found that the expression levels of KIF15 were significantly downregulated in B7-H3-knockdown RKO cells after 4 Gy X-ray irradiation via RNA-seq analysis. Moreover, the protein level of KIF15 was upregulated when B7-H3 was overexpressed, while the protein level of KIF15 was downregulated in B7-H3-knockdown CRC cells after 4 Gy X-ray irradiation. Importantly, both KIF15 knockdown and treatment with SB743921, a KIF15-specific inhibitor, reversed the B7-H3-induced radioresistant effect in CRC in vitro and in vivo. In addition, we first reported that KIF15 was upregulated in CRC tissue samples compared with normal adjacent tissues and positively correlated with TNM stage. More importantly, there was a positive correlation between the levels of B7-H3 and KIF15 in CRC patients. These results suggest that B7-H3 promotes CRC radioresistance via KIF15.

Studies have found that NF-κB is widely present in a variety of cells and induces high expression of inflammatory factors, which is involved in antigen-antibody immune response, inflammation response, cell proliferation and apoptosis^33^. Studies have shown that solar ultraviolet radiation (UVR) significantly increases the expression of PD-L1 molecules in melanocytes, and melanocytes and keratinocytes secrete HMGB1, thereby promoting PD-L1 dependence in melanocytes NF-κB and IRF3 transcription^34^. Western blot assay found that B7-H3, which was up-regulated after X-ray irradiation, could regulate the expression level of KIF15 through NF-κB. Our previous research also found that B7-H3 can promote the activity of NF-κB promoter^16^. However, we have yet fully understood the specific mechanism by which B7-H3 activates the NF-κB pathway. Therefore, the manner in which B7-H3 regulates NF-κB requires further exploration in future research.

The ERK signaling pathway is well known to play a critical role in several cell processes, including cell cycle progression, cell proliferation, transcription, survival and apoptosis^35^. Above all, the aberrant activation of ERK signaling is involved in radioresistance in several cancers^15^. The MEK/ERK pathway plays a key role in sustaining the tumorigenicity and in vitro radioresistance of the embryonal rhabdomyosarcoma stem-like cell population^36^. In addition, the ERK pathway has been reported to be involved in mitochondrial ATP-sensitive potassium channel-mediated glioma radioresistance^37^. Consistent with previous results^24,38^, our luciferase reporter assay showed that the B7-H3/KIF15 axis significantly increased ERK activity in CRC cells after 4 Gy X-ray irradiation. Western blot and immunofluorescence assays further confirmed the luciferase reporter results. Moreover, treatment with PD98059, an ERK inhibitor, abolished B7-H3-induced radioresistance in CRC cells. Therefore, our results suggest that the B7-H3/KIF15 axis protects CRC cells from X-ray irradiation by activating the ERK signaling pathway.

In conclusion, we illustrated that the upregulation of B7-H3 in CRC cells after X-ray irradiation contributes to CRC radioresistance via the KIF15/ERK signaling pathway. Moreover, high KIF15 expression is positively correlated with B7-H3 and TNM stages in CRC tissue samples. Additionally, B7-H3 blockade by 3E8 combined with irradiation significantly controlled tumor growth in xenograft tumor model. Overall, our results indicate that B7-H3 is a potential biomarker that can be used to identify responders for radiotherapy and that the combination of B7-H3 blockade and radiotherapy may improve the therapeutic regimen against CRC.

## Supplementary information

Supplementary information

Figure S1

Figure S2

Figure S3

Figure S4

Figure S5

Figure S6
