# Supplementary material for: B7-H3 regulates KIF15-activated ERK1/2 pathway and contributes to radioresistance in colorectal cancer
Source: Cell Death Dis. 2020 Oct 3;11(10):824. doi: 10.1038/s41419-020-03041-4 (PMC7532977; doi:10.1038/s41419-020-03041-4)
Supplement: Supplementary file 1 — Supplementary information [file 41419_2020_3041_MOESM1_ESM.docx]

**Supplementary information to:**

**B7-H3 regulates KIF15-activated ERK1/2 pathway and contributes to radioresistance in colorectal cancer**

**Materials and Methods**

**Cell transfection and lentivirus infection**

Lentivirus vectors carrying human B7-H3 cDNA and B7-H3 shRNA were generated by Genechem Co., Ltd. (Shanghai, China). An empty backbone vector was used as a control. For lentivirus infection, HCT116 and RKO cells at 30% confluence in 6-well plates were transduced with lentiviral particles at an MOI of 20. The infection efficiency was confirmed by counting GFP-expressing cells under a fluorescence microscope 72 h after infection. The expression plasmids carrying human B7-H3 cDNA and its control plasmids were purchased from GenePharma Co., Ltd. (Shanghai, China). Commercial KIF15 siRNA and its control siRNA were purchased from RIBOBIO (Guangzhou, China). HCT116 and RKO cells were transfected with siRNA reagents using Lipofectamine 2000 (Invitrogen, CA, USA) according to the manufacturer’s instructions. Transfection efficiency was determined by RT-qPCR and Western blot assays.

**RNA isolation and RT-qPCR**

Total RNA from cultured cells was prepared using TRIzol reagent (TaKaRa, Shiga, Japan) according to the manufacturer’s instructions. A total of 1 μg of RNA was reverse-transcribed using a cDNA Reverse Transcription Kit (TaKaRa) according to the manufacturer’s instructions. The acquired cDNA was analyzed in triplicate by real-time PCR on a CFX96 Touch^TM^ Real-Time PCR system (Bio-Rad, CA, USA) using a SYBR PrimeScript RT-qPCR Kit (Takara). The PCR conditions were as follows: 95 °C for 5 min, followed by 40 cycles of amplification for 30 s at 95 °C, 45 s at 60 °C and 45 s at 72 °C. Individual gene expression was normalized to that of β-actin mRNA. The primer sequences for RT-qPCR are provided in Supplementary materials, Table S3.

**Protein extraction and Western blot analysis**

HCT116 or RKO cells in 6-well plates were lysed with RIPA lysis buffer (Thermo Scientific, Waltham, MA, USA) containing a protease inhibitor cocktail (Sigma, St. Louis, Missouri, USA) according to the manufacturer’s instructions. Protein concentrations were measured with a Pierce BCA protein assay kit (Thermo Scientific). Equal amounts of protein were separated by 10% SDS-PAGE and transferred to a PVDF membrane (Merck Millipore, Darmstadt, Germany). The antibodies used for western blot analysis in this study were as follows: goat anti-human B7-H3 (R&D Systems, #AF1027), rabbit anti-human KIF15 (Proteintech, #55407-1-AP), rabbit anti-human ERK (CST, #4695), rabbit anti-human p-ERK (CST, #4370), anti-human Bcl-2 (Abcam, #ab32124), rabbit anti-human/mouse Bax (Abcam, #ab32503), rabbit anti-human Caspase 3 (CST, #9661), rabbit anti-human Cyclin B1 (CST, #12231), rabbit anti-human CDK1 (CST, #9116) and mouse anti-human/mouse β-actin (CST, #3700). The membranes were developed with Clarity Western ECL substrate (Bio-Rad, CA, USA) and visualized with a ChemiDoc^TM^ MP imaging system (Bio-Rad). The band density was analyzed using Image J software.

**Colony formation assays**

Cells were plated into 12-well plates and then exposed to 0, 2, 4, 6 or 8 Gy irradiation. Cells were exposed to different doses of ionizing radiation using an X-ray linear accelerator (RadSource, Suwanee, GA, USA) at a fixed dose rate of 2 Gy/min. The colonies were fixed with methanol for 30 min and then stained with 1% crystal violet (Sigma) for 30 min. The number of colonies (≥50 cells per colony) was counted under a light microscope. Plating efficiency (PE) (%) = number of colonies/original cell-seeding density × 100%, and surviving fraction (SF) = number of colonies/(number of cells seeded × PE). The cell survival curve was mesured using the multi-target single-hit model: SF=1-(1-e^-D/D0^)^N^, and the radiobiological parameters D0 (the dose that gave an average of one hit per target), Dq (quasi-threshold dose), K (cell inactivation constant) and N (extrapolation number) were determined using the survival curve. Additionally, the radiation sensitivity enhancement ratio (SER) = D0 of the blank or negative control group/D0 of the transfection group. All experiments were conducted in quintuplicate.

**CCK8 assay**

Cells were seeded into 96-well plates at an initial density of 5*10^3^ cells per well for 24 h and irradiated at a dose of 4 Gy. Then, the cells were stained with 10 μl of sterile CCK8 (Dojindo Laboratory, Mashikimachi, Japan) for 4 h at 37 °C. The absorbance at 450 nm was used as the reference wavelength.

**Flow cytometry**

Cells were harvested and washed with cold PBS, and the cell cycle distribution and apoptosis rate were analyzed by flow cytometry (Beckman Coulter, CA, USA). Briefly, CRC cells were plated into 6-well plates at 10^5^ cells per well for 24 h. Then, the cells were exposed to 4 Gy. To analyze the cell cycle distribution, cells were collected after 24 h, washed twice with PBS, fixed in 70% ethanol containing 0.5% FBS and stored at -20 °C for at least 24 h. Samples were washed twice with PBS, treated with cell cycle reagent (Fcmrcs, Nanjing, China), and analyzed by flow cytometry using ModFit LT 3.1. For apoptosis analysis, the cells were washed with cold PBS and stained with Annexin V-PE and 7-AAD (BD, Franklin Lakes, NJ, USA) according to the manufacturer’s instructions. Annexin-V+/7-AAD- cells and Annexin-V+/7-AAD+ cells were considered apoptotic cells.

**Xenograft tumor model**

Female nude mice (6-8 weeks, 18-20 g) were purchased from the Shanghai Laboratory Animal Center. All mice were synchronized with a 12 h light/dark cycle in an autonomous chronobiological animal facility (Suzhou, China), with the lights on from 6 am (Zeitgeber time 0) to 6 pm (Zeitgeber time 12) for 1 week. All experimental procedures were approved by the Institutional Animal Care and Use Committee of Soochow University (Suzhou, China). Three *in vivo* experiments were designed. In experiment 1, the mice were divided randomly into the HCT116-EV (empty vector, EV)+0 Gy (0 Gy), HCT116-EV+6 Gy (6 Gy) and HCT116-EV+10 Gy (10 Gy) groups, and equal amounts of HCT116 cells (5*10^6^) were injected subcutaneously into the flank of each mouse. The mice in the HCT116-EV+6 Gy (6 Gy) and HCT116-EV+10 Gy (10 Gy) groups were irradiated at a single dose of 6 or 10 Gy X-rays from a linear accelerator (Varian) at a dose rate of 0.5 Gy/min on day 6. In experiment 2, the mice were randomly divided into HCT116-EV (EV)+IgG antibody (EV+IgG), HCT116-EV (EV)+B7-H3 antibody (EV+Ab), HCT116-B7-H3+IgG antibody (B7H3+IgG) and HCT116-B7-H3+B7-H3 antibody (B7H3+Ab) groups (n=5 per group), and equal amounts of HCT116-B7-H3 cells (5*10^6^) were injected subcutaneously into the flank of each mouse. 3E8 (5 mg/kg, Bright Scistar Biotechnology Co., Ltd., Suzhou, China), a B7-H3 blocking antibody, or IgG was given intraperitoneally to mice in the EV/B7H3+Ab group every other day for 2 weeks. 3E8/IgG treatment began on day 6. In experiment 3, the mice were divided randomly into the HCT116-EV+DMSO (EV+DMSO), HCT116-B7H3+DMSO (B7H3+DMSO) and HCT116-B7-H3+SB743921 (B7-H3+SB743921) groups (n=5 per group), and equal amounts of HCT116-B7-H3 or control cells (5*10^6^) were injected subcutaneously into the flank of each mouse. The mice in the B7-H3+SB743921 or EV/B7H3+DMSO group were intraperitoneally injected with SB743921 (2.5 mg/kg) or vehicle control (DMSO) on day 6^1^. All mice in experiments 2 and 3 were irradiated at a single dose of 10 Gy X-rays from a linear accelerator at a dose rate of 0.5 Gy/min on day 12. The tumors were examined twice weekly; the length and width measurements were obtained with calipers, and the tumor size was calculated. On day 21, the animals were euthanized, and the tumors were excised and weighed. Tumor size (mean ± SEM; mm^2^) was calculated according to the following equation: tumor size (mm^2^) =S (mm) × L (mm), where S and L are the smallest and largest perpendicular tumor diameters, respectively^2^.

**TUNEL assay**

For the apoptosis assay, the xenografted tumor tissues of nude mice were examined using an *in situ* Cell Death Detection Kit (Roche Diagnostic, Mannheim, Germany) according to the manufacturer’s instructions. Briefly, sections from paraffin-embedded xenograft tumor tissues were dewaxed and rehydrated and then incubated with TUNEL reaction mixture at 37 °C for 1 h in a chamber with a humidified atmosphere. Nuclei were stained with DAPI. The numbers of TUNEL-positive cells and total cells were analyzed using a confocal microscope.

**RNA sequencing analysis**

sh-NC or sh-B7-H3-RKO cells were seeded into 6-well plates for 24 h and irradiated at a dose of 4 Gy. After 24 h, total RNA was collected for each group. The microarray analysis was carried out by GENEWIZ Biotech Co. (Suzhou, China).

**Luciferase assay**

The ERK (Elk-1/SRF)-responsive luciferase reporter was purchased from Genomeditech (#GM-021045, Shanghai, China). Luciferase activity was measured according to the manufacturer’s instructions (Promega, E1910, Fitchburg, USA).

**Immunofluorescence**

The human B7-H3 plasmid and its control plasmid were purchased from GenePharma Co., Ltd. (Shanghai, China). HCT116 and RKO cells were transfected with plasmid reagents using Lipofectamine 2000 (Invitrogen) according to the manufacturer’s instructions. Transfection efficiency was determined by Western blot assays. Cells were washed with PBS, fixed with 4% paraformaldehyde and blocked with 5% BSA/PBS for 30 min at 37 °C. Then, primary antibodies, rabbit anti-human KIF15 (Proteintech, 1:200) and mouse anti-human p-ERK (CST, #5726, 1:200), were incubated with cells overnight at 4 °C, after which a Cy3-conjugated mouse (Abcam, ab97035, 1:500) and FITC-conjugated rabbit (Thermo Fisher, F-2765, 1:1000) secondary antibody was incubated with the cells for 1 h at 37 °C. The cells were counterstained with DAPI to visualize the nuclei and examined using a confocal microscope (Zeiss, Oberkochen, German).

**Patients and samples**

Between April 2010 and February 2014, 123 pairs of CRC tissue samples and the corresponding normal adjacent tissue samples were obtained from surgical procedures from the First Affiliated Hospital of Soochow University (Suzhou, China) with the consent of all patients. This study was approved by the Ethical Committee of Soochow University. Clinical pathological characteristics, including age, sex and TNM stage, were recorded (Supplementary materials, Table S2).

**Table S1. RNA-seq results**

Downregulated Genes

| Gene ID | Gene Name | Fold Change | P Value |
| --- | --- | --- | --- |
| ENSG00000129195 | FAM64A | -14.341 | 0.0343 |
| ENSG00000111912 | NCOA7 | -14.1361 | 0.0488 |
| ENSG00000118257 | NRP2 | -14.1084 | 0.04235 |
| ENSG00000168591 | TMUB2 | -14.0462 | 0.04165 |
| ENSG00000141956 | PRDM15 | -13.8905 | 0.02415 |
| ENSG00000163808 | KIF15 | -13.0755 | 0.04505 |
| ENSG00000170581 | STAT2 | -13.0286 | 0.0392 |
| ENSG00000102977 | ACD | -12.9751 | 0.0413 |
| ENSG00000106799 | TGFBR1 | -12.8594 | 0.04045 |
| ENSG00000129667 | RHBDF2 | -12.7612 | 0.03895 |
| ENSG00000174405 | LIG4 | -12.5814 | 0.03785 |
| ENSG00000151612 | ZNF827 | -12.4511 | 0.04345 |
| ENSG00000196843 | ARID5A | -12.3276 | 0.03855 |
| ENSG00000106268 | NUDT1 | -12.2867 | 0.04855 |
| ENSG00000176208 | ATAD5 | -11.874 | 0.04715 |
| ENSG00000108423 | TUBD1 | -11.168 | 0.04775 |
| ENSG00000103855 | CD276 | -6.26791 | 0.0002 |
| ENSG00000140848 | CPNE2 | -4.13993 | 0.04935 |
| ENSG00000103855 | CD276 | -3.66595 | 0.0043 |
| ENSG00000198444 | F8A2 | -3.33373 | 0.02165 |
| ENSG00000115594 | IL1R1 | -3.32659 | 0.0429 |
| ENSG00000176658 | MYO1D | -3.21489 | 0.03035 |
| ENSG00000213860 | RPL21P75 | -3.20175 | 5.00E-05 |
| ENSG00000101849 | TBL1X | -3.03101 | 0.03195 |
| ENSG00000165949 | IFI27 | -2.83061 | 0.03675 |
| ENSG00000114315 | HES1 | -2.80901 | 0.0267 |
| ENSG00000099875 | MKNK2 | -2.73848 | 0.03945 |
| ENSG00000168056 | LTBP3 | -2.60405 | 0.0384 |
| ENSG00000102384 | CENPI | -2.37569 | 0.0321 |
| ENSG00000196550 | FAM72A | -2.34873 | 0.03355 |
| ENSG00000153823 | PID1 | -2.24414 | 0.0289 |
| ENSG00000173517 | PEAK1 | -2.24375 | 0.03405 |
| ENSG00000168159 | RNF187 | -2.20669 | 0.0041 |
| ENSG00000180998 | GPR137C | -2.16917 | 0.03735 |
| ENSG00000162704 | ARPC5 | -2.15429 | 0.02125 |
| ENSG00000218336 | TENM3 | -2.14724 | 0.01705 |
| ENSG00000035499 | DEPDC1B | -2.10479 | 5.00E-05 |
| ENSG00000087842 | PIR | -2.08575 | 0.01935 |

Upregulated Genes

| Gene ID | Gene Name | Fold Change | P Value |
| --- | --- | --- | --- |
| ENSG00000176928 | GCNT4 | 2.01839 | 0.0419 |
| ENSG00000236453 | AC003092.1 | 2.02083 | 0.0158 |
| ENSG00000125398 | SOX9 | 2.02683 | 0.00125 |
| ENSG00000225138 | CTD-2228K2.7 | 2.03054 | 0.03985 |
| ENSG00000033867 | SLC4A7 | 2.04215 | 5.00E-05 |
| ENSG00000118473 | SGIP1 | 2.05263 | 0.042 |
| ENSG00000145390 | USP53 | 2.07705 | 0.01485 |
| ENSG00000074416 | MGLL | 2.09373 | 0.0142 |
| ENSG00000109046 | WSB1 | 2.14767 | 0.0011 |
| ENSG00000010818 | HIVEP2 | 2.15635 | 0.01115 |
| ENSG00000165102 | HGSNAT | 2.16341 | 0.00515 |
| ENSG00000131016 | AKAP12 | 2.16696 | 5.00E-05 |
| ENSG00000100888 | CHD8 | 2.17001 | 0.04835 |
| ENSG00000143772 | ITPKB | 2.17534 | 0.0376 |
| ENSG00000078177 | N4BP2 | 2.17681 | 5.00E-05 |
| ENSG00000178104 | PDE4DIP | 2.18157 | 0.0125 |
| ENSG00000117226 | GBP3 | 2.24753 | 0.03935 |
| ENSG00000244257 | PKD1P1 | 2.35639 | 0.02485 |
| ENSG00000169446 | MMGT1 | 2.40145 | 0.0397 |
| ENSG00000110841 | PPFIBP1 | 2.42564 | 0.0105 |
| ENSG00000140181 | HERC2P2 | 2.51751 | 0.01205 |
| ENSG00000058085 | LAMC2 | 2.52385 | 0.0168 |
| ENSG00000177409 | SAMD9L | 2.61112 | 0.02435 |
| ENSG00000149311 | ATM | 2.68186 | 0.01405 |
| ENSG00000235173 | FAM203A | 2.69342 | 0.0144 |
| ENSG00000002079 | MYH16 | 2.81045 | 0.01215 |
| ENSG00000238103 | RPL9P7 | 2.81079 | 0.00025 |
| ENSG00000144583 | 4-Mar | 3.05527 | 0.00025 |
| ENSG00000138356 | AOX1 | 3.08785 | 5.00E-05 |
| ENSG00000161048 | NAPEPLD | 3.09873 | 0.04975 |
| ENSG00000185990 | F8A3 | 3.21716 | 0.00185 |
| ENSG00000138356 | AOX1 | 3.24753 | 0.0009 |
| ENSG00000078177 | N4BP2 | 3.34174 | 0.00835 |
| ENSG00000150990 | DHX37 | 3.47741 | 0.01755 |
| ENSG00000229563 | RP11-245M24.1 | 3.89758 | 0.0016 |
| ENSG00000196611 | MMP1 | 4.13638 | 5.00E-05 |
| ENSG00000166670 | MMP10 | 5.16891 | 0.01635 |
| ENSG00000077238 | IL4R | 11.811 | 0.0491 |
| ENSG00000164088 | PPM1M | 11.9501 | 0.0464 |
| ENSG00000006432 | MAP3K9 | 11.9589 | 0.04865 |
| ENSG00000136104 | RNASEH2B | 12.3184 | 0.0448 |
| ENSG00000173918 | C1QTNF1 | 12.5049 | 0.0309 |
| ENSG00000214113 | LYRM4 | 12.6564 | 0.04695 |
| ENSG00000106268 | NUDT1 | 12.6973 | 0.04075 |
| ENSG00000204574 | ABCF1 | 12.84 | 0.0486 |
| ENSG00000107960 | OBFC1 | 12.8946 | 0.04965 |
| ENSG00000175066 | GK5 | 13.3535 | 0.03985 |
| ENSG00000136682 | CBWD2 | 13.3885 | 0.0373 |
| ENSG00000125814 | NAPB | 13.5746 | 0.0392 |
| ENSG00000164074 | C4orf29 | 13.6359 | 0.02945 |
| ENSG00000113763 | UNC5A | 13.8385 | 0.04275 |
| ENSG00000164181 | ELOVL7 | 13.8932 | 0.03215 |
| ENSG00000182798 | MAGEB17 | 13.9418 | 0.0406 |
| ENSG00000181894 | ZNF329 | 14.1004 | 0.0302 |
| ENSG00000106459 | NRF1 | 14.138 | 0.0326 |
| ENSG00000165752 | STK32C | 14.3664 | 0.04575 |
| ENSG00000183506 | PI4KAP2 | 14.4535 | 0.0387 |
| ENSG00000115594 | IL1R1 | 15.4912 | 0.0256 |

**Table S2. Clinical characteristics of patients**

| CRC patients | Number | B7-H3 expression  P value | KIF15 expression  P value |
| --- | --- | --- | --- |
| No. of patients | 123 |  |  |
| Gender |  | 0.3425 | 0.6955 |
| Male | 72 |  | |
| Female | 51 |  | |
| Age (years) |  | 0.5996 | 0.1059 |
| Mean | 60.42 |  | |
| Range | 26-81 |  | |
| Tumor location |  | 0.01 | 0.1823 |
| Colon | 72 |  | |
| Rectum | 51 |  | |
| TNM stage |  | < 0.0001 | < 0.0001 |
| I-II | 64 |  | |
| III-IV | 59 |  | |

**Table S3. Primers for RT-qPCR assay of gene**

| Primer Name | Primer Sequence (5’-3’) |
| --- | --- |
| Homo-B7-H3 Forward | ACAGGGCAGCCTATGACATT |
| Homo-B7-H3 Reverse | CTGCATTCTCCTCCTCACAG |
| Homo-IL1R1 Forward | ATGAAATTGATGTTCGTCCCTGT |
| Homo-IL1R1 Reverse | ACCACGCAATAGTAATGTCCTG |
| Homo-NRF1 Forward | AGGAACACGGAGTGACCCAA |
| Homo-NRF1 Reverse | TATGCTCGGTGTAAGTAGCCA |
| Homo-STK32C Forward | CATGGTCGTGGACCTGCTAC |
| Homo-STK32C Reverse | ACCGTGTCCTCGGAGAACT |
| Homo-ZNF329 Forward | AAATGACGACTCGGAATTTTCCT |
| Homo-ZNF329 Reverse | AAAGCTGATTGCCTCAAGTGT |
| Homo-UNC5A Forward | GGACACCCGCAACTGTACC |
| Homo-UNC5A Reverse | AATGGACGAGTCAGCCACATC |
| Homo-FAM64A Forward | CCTGGAAACGCCTGGAAAC |
| Homo-FAM64A Reverse | CAAAGCACTCTTAGCTGAGCG |
| Homo-NCOA7 Forward | AAAGACGCTCTACCGGAAATCG |
| Homo-NCOA7 Reverse | CGCCTGTGCCATAATAGTGGT |
| Homo-TMUB2 Reverse | TGAGGGTGTGGGTAATGAGGT |
| Homo-TMUB2 Reverse | AGAGAGCCAAGCTAGGACCAA |
| Homo-KIF15 Reverse | AAAACTGAGTTACGCAGCGTG |
| Homo-KIF15 Reverse | AGTTGCGAATACAGATTCCTGAG |
| Homo-PRDM15 Reverse | TGAAGATGGGAGCGAAGAGAT |
| Homo-PRDM15 Reverse | ACCTTGCCCTGCTTAACACAA |
| Homo-β-actin Forward | CATGTACGTTGCTATCCAGGC |
| Homo-β-actin Reverse | CTCCTTAATGTCACGCACGAT |

**Supplementary Figure Legends**

**Fig. S1.** B7-H3 contributes to radioresistance of colorectal cells *in vitro*.

**A** B7-H3 protein levels in NCM460, RKO, HCT116, HT29, HCT8, SW480 and SW620 cells were analyzed by Western blot. β-actin served as a loading control. Values are expressed as the mean ± SD. **B**, **C** B7-H3 mRNA and protein levels in B7-H3-overexpressing HCT116 and RKO cells were analyzed by RT-qPCR **B** and Western blot **C**. β-actin served as a loading control. **D**, **E** B7-H3 mRNA and protein levels in B7-H3-knockdown HCT116 and RKO cells were analyzed by RT-qPCR **D** and Western blot **E**. β-actin served as a loading control. Values are expressed as the mean ± SD. **F** The effect of B7-H3 knockdown on the colony formation of HCT116 and RKO cells after 4 Gy X-ray irradiation. *P<0.05, **P<0.01.

**Fig. S2.** B7-H3 decreases the cell cycle arrest and cell apoptosis of CRC cells.

**A**, **B** The protein expression of CDK1 and Cyclin B1 in B7-H3-overexpressing **A** or B7-H3-knockdown **B** CRC cells after exposure to 4 Gy X-ray irradiation. β-actin served as a loading control. Values are expressed as the mean ± SD. **C**, **D** The relative protein expression of Bcl-2, Bax and cleaved-caspase 3 in B7-H3-overexpressing **C** or B7-H3-knockdown **D** CRC cells after exposure to 4 Gy X-ray irradiation. β-actin served as a loading control. Values are expressed as the mean ± SD. *P<0.05, **P<0.01.

**Fig. S3.** B7-H3 contributes to radioresistance of CRC *in vitro* and *in vivo*.

**A** Viability was assessed in EV or B7-H3-overexpressing HCT116 and RKO cells treated with different concentrations of 3E8 prior to treatment with 4 Gy X-ray irradiation. **B** Representative images of tumors formed by HCT116 cells treated with 0, 6 or 10 Gy X-ray irradiation. **C** The growth curves of tumors formed by HCT116 cells treated with 0, 6 or 10 Gy X-ray irradiation. The data are presented as the mean ± SD. **D** The weights of tumors formed by the indicated HCT116 cells treated with 0, 6 or 10 Gy X-ray irradiation. The data are presented as the mean ± SD. n=3-4. ns, not significant. *P<0.05, **P<0.01.

**Fig.** **S4.** B7-H3 promotes radioresistance via KIF15 *in vitro*.

**A** The mRNA levels of IL1R1, NRF1, STK32C, ZNF329, UNC5A, FAM64A, NCOA7, TMUB2, KIF15 and PRDM15 were analyzed in sh-B7-H3 and sh-NC RKO cells by RT-qPCR. **B** The relative protein expression of KIF15 in B7-H3-overexpressing or B7-H3-knockd own CRC cells after exposure to 4 Gy X-ray irradiation. β-actin served as a loading control. Values are expressed as the mean ± SD. **C**, **D** KIF15 mRNA **C** and protein **D** levels in KIF15-knockdown HCT116 and RKO cells were analyzed by Western blot and RT-qPCR. β-actin served as a loading control. **E** Representative colonies from EV/B7-H3-CRC cells treated with KIF15 siRNA or SB743921 prior to treatment with 4 Gy X-ray irradiation. **F** Apoptosis was measured using Annexin V/7-AAD double staining in B7-H3-CRC cells treated with KIF15 siRNA or SB743921 prior to treatment with 4 Gy X-ray irradiation. *P<0.05, **P<0.01.

**Fig.** **S5.** B7-H3 promotes radioresistance via KIF15 *in vitro* and *in vivo*.

**A** Western blot was performed to investigate Bcl-2, Bax and cleaved-caspase 3 expression in B7-H3-overexpressing HCT116 and RKO cells treated with KIF15 siRNA prior to 4 Gy X-ray irradiation. β-actin served as a loading control. Values are expressed as the mean ± SD. **B** The effect of B7-H3 overexpression on cell cycle progression in HCT116 and RKO cells treated with KIF15 siRNA or SB743921 prior to treatment with 4 Gy X-ray irradiation. **C** Western blot was performed to investigate CDK1 and Cyclin B1 expression in B7-H3-overexpressing HCT116 and RKO cells treated with KIF15 siRNA prior to 4 Gy X-ray irradiation. β-actin served as a loading control. Values are expressed as the mean ± SD. **D** KIF15 and Ki67 IHC staining in tumor tissues of the xenograft model with the indicated treatments (scale bar, 50 μm). **E** TUNEL staining in tumor tissues of the xenograft model with the indicated treatments (scale bar, 50 μm). *P<0.05, **P<0.01.

**Fig. S6.** B7-H3 inhibited cell apoptosis via KIF15/ERK.

**A** The relative protein expression of p-ERK and ERK in B7-H3-overexpressing CRC cells treated with KIF15 siRNA prior to treatment with 4 Gy X-ray irradiation. β-actin served as a loading control. Values are expressed as the mean ± SD. **B** B7-H3 protein levels following transfection with the B7-H3 plasmid in HCT116 and RKO cells were analyzed by Western blot. β-actin served as a loading control. **C** Apoptosis was measured using Annexin V/7-AAD double staining in B7-H3-CRC cells treated with PD98059 prior to treatment with 4 Gy X-ray irradiation. **D** Western blot was performed to investigate cleaved-caspase 3 expression in B7-H3-overexpressing HCT116 and RKO cells treated with PD98059 prior to 4 Gy X-ray irradiation. β-actin served as a loading control. Values are expressed as the mean ± SD. *P<0.05, **P<0.01.

**References**

1. Yue Yin, et al. Kinesin spindle protein inhibitor SB743921 induces mitotic arrest and apoptosis and overcomes imatinib resistance of chronic myeloid leukemia cells. *Leuk. Lymphoma* **56**, 1813-1820 (2015).

2. Yang, H., et al. STAT3 Inhibition Enhances the Therapeutic Efficacy of Immunogenic Chemotherapy by Stimulating Type 1 Interferon Production by Cancer Cells. *Cancer Res.* **75**, 3812-3822 (2015).
