# Supplementary figures and images for: B7-H3 regulates KIF15-activated ERK1/2 pathway and contributes to radioresistance in colorectal cancer
Source: Cell Death Dis. 2020 Oct 3;11(10):824. doi: 10.1038/s41419-020-03041-4 (PMC7532977; doi:10.1038/s41419-020-03041-4)

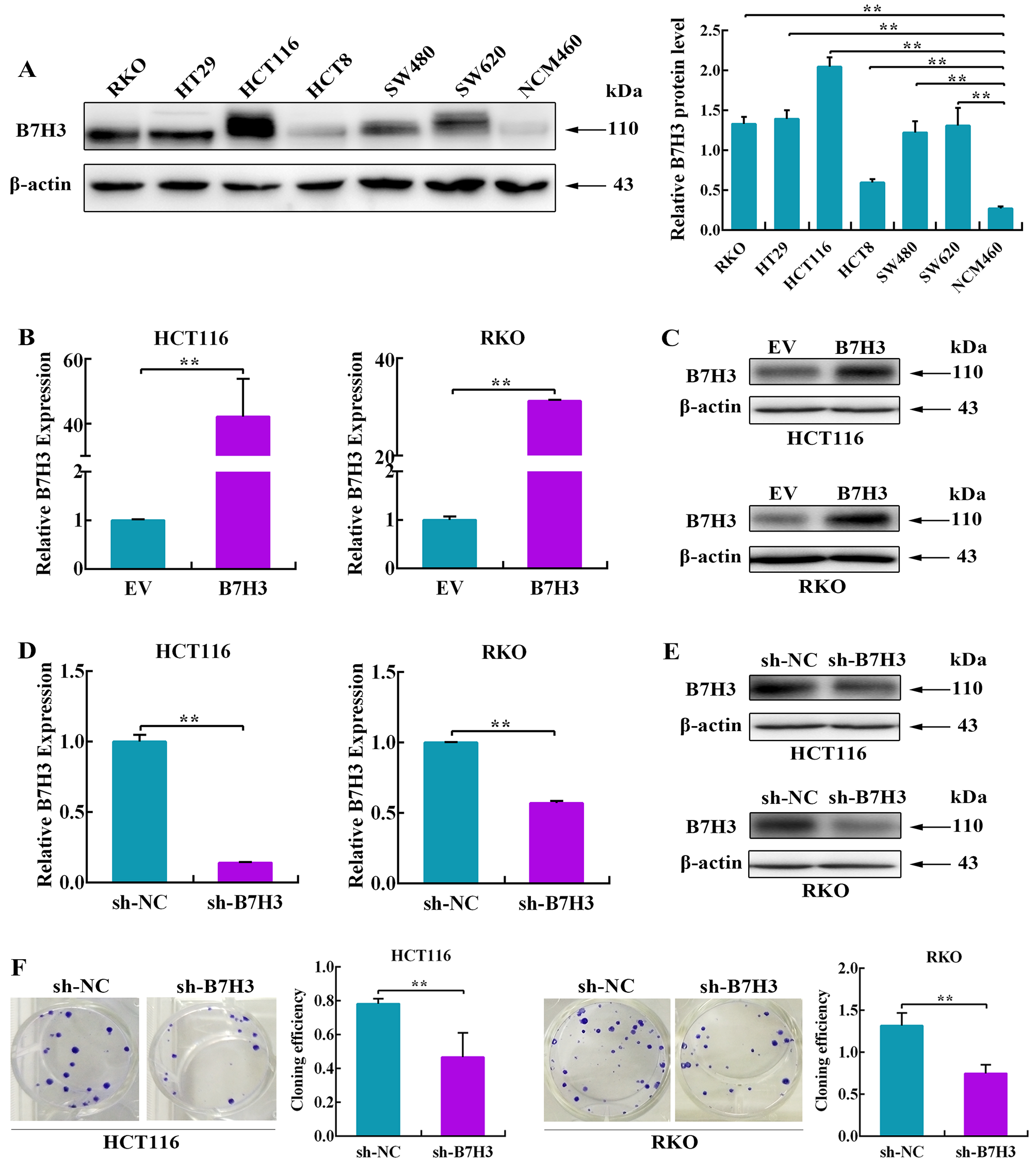

Supplement: Supplementary file 2 — Figure S1 [file 41419_2020_3041_MOESM2_ESM.tif]

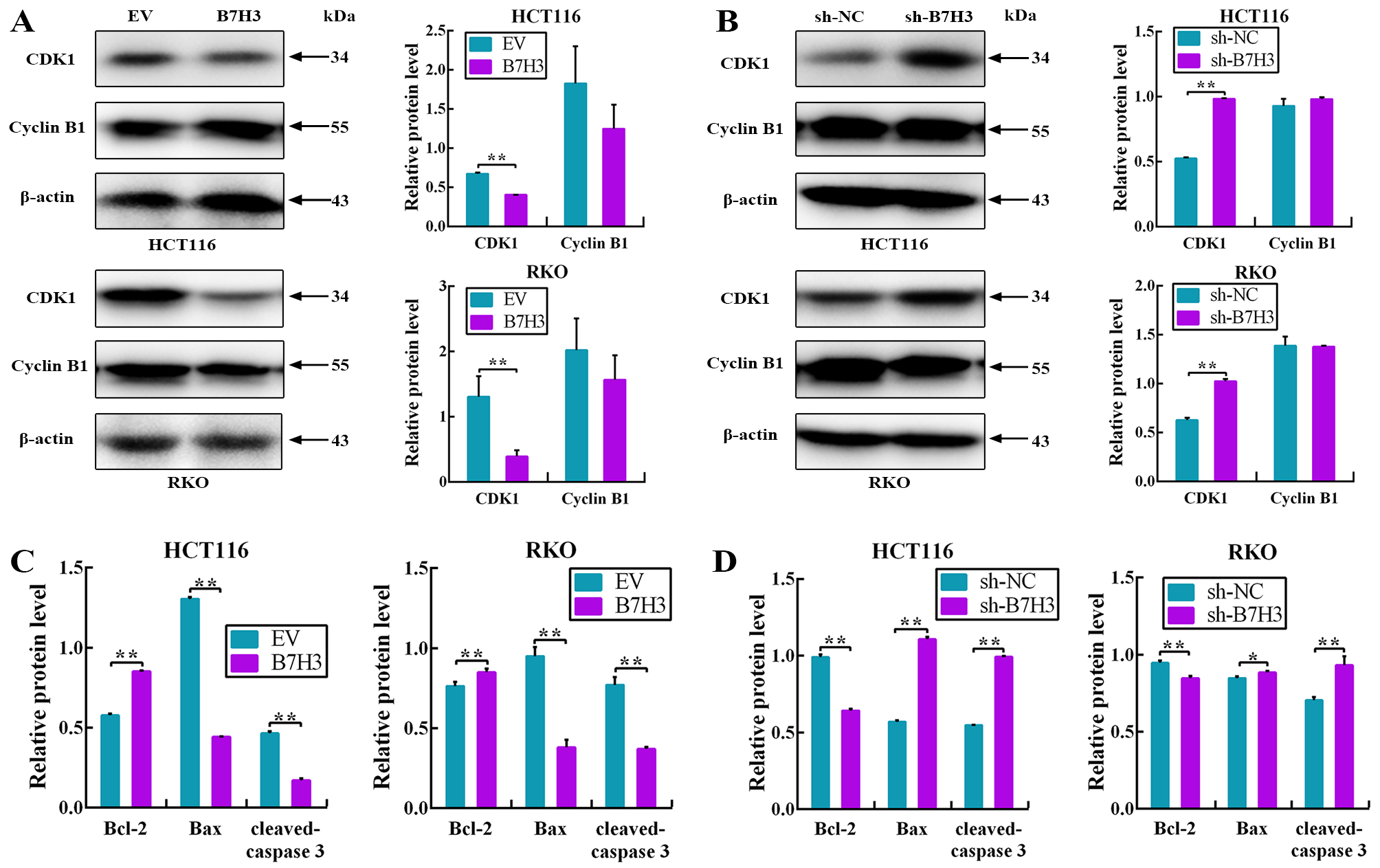

Supplement: Supplementary file 3 — Figure S2 [file 41419_2020_3041_MOESM3_ESM.tif]

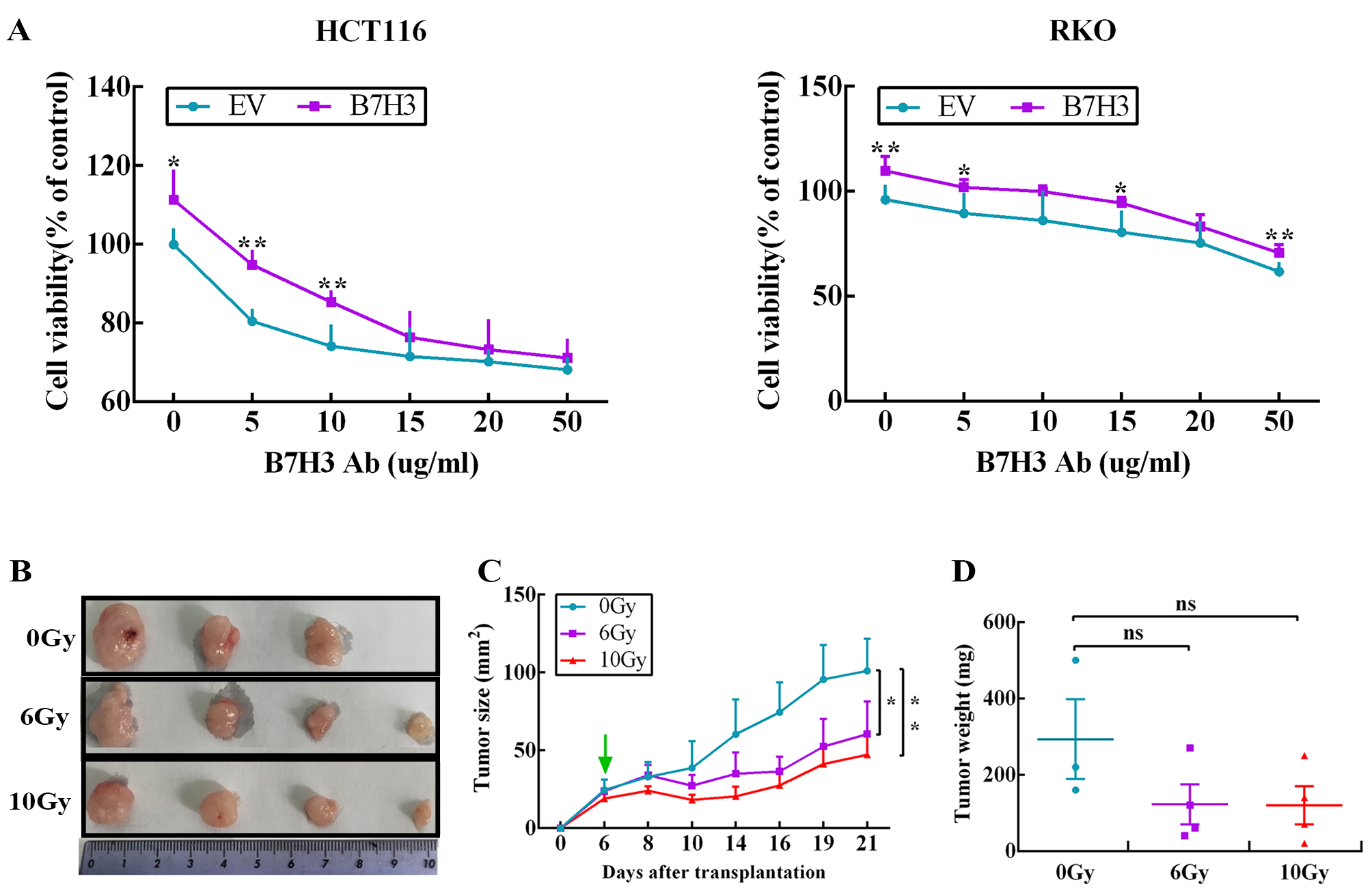

Supplement: Supplementary file 4 — Figure S3 [file 41419_2020_3041_MOESM4_ESM.tif]

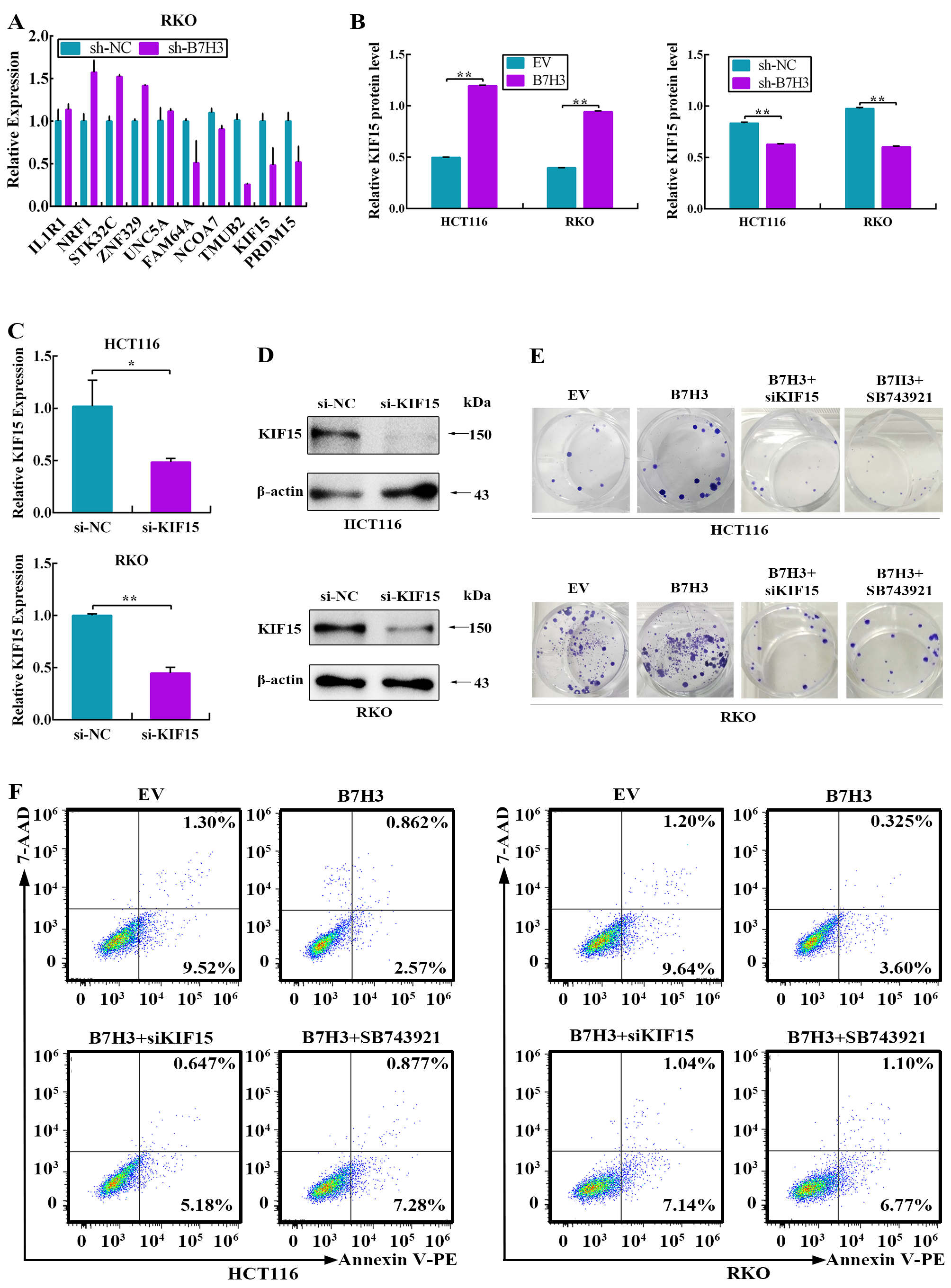

Supplement: Supplementary file 5 — Figure S4 [file 41419_2020_3041_MOESM5_ESM.tif]

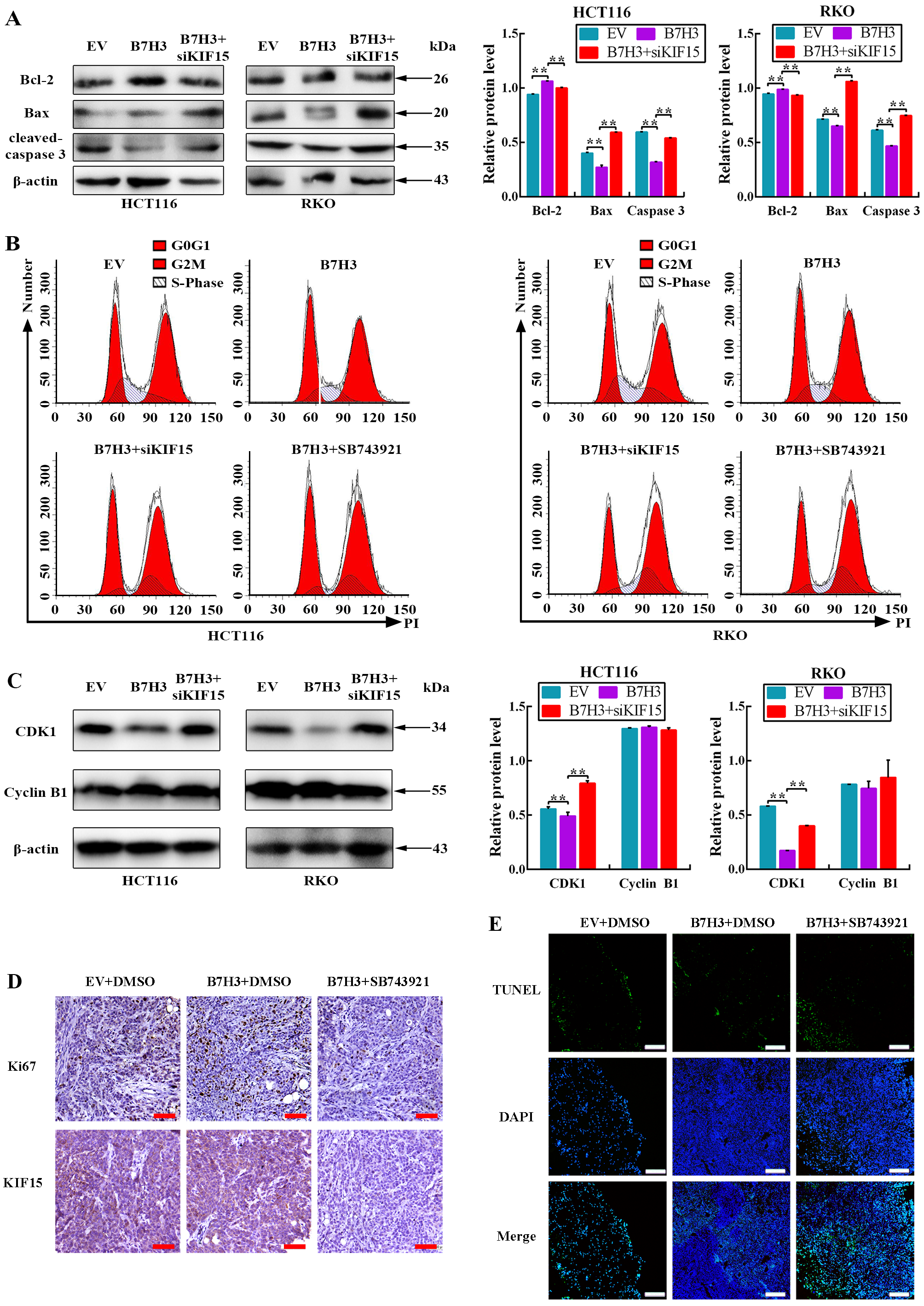

Supplement: Supplementary file 6 — Figure S5 [file 41419_2020_3041_MOESM6_ESM.tif]

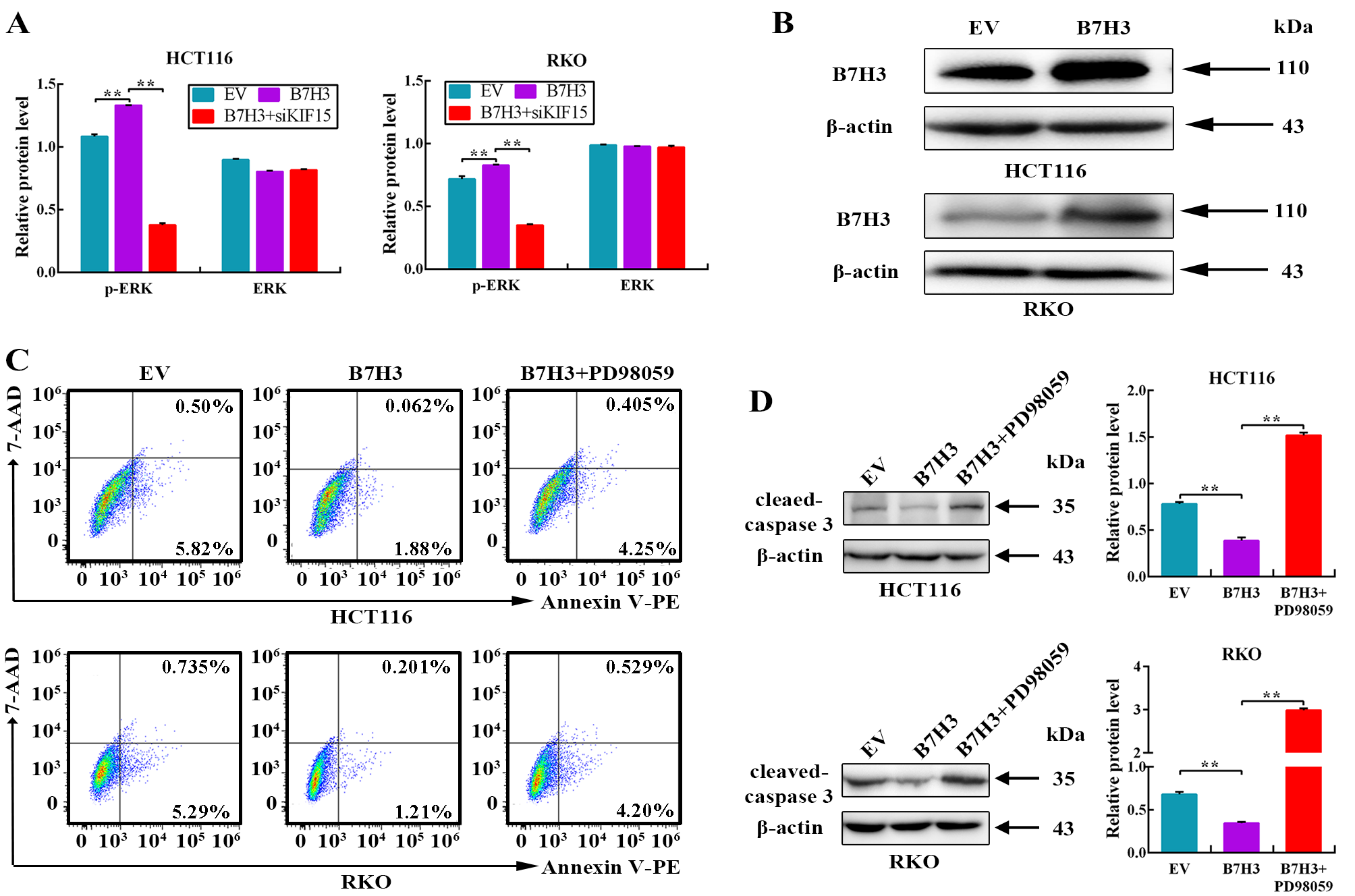

Supplement: Supplementary file 7 — Figure S6 [file 41419_2020_3041_MOESM7_ESM.tif]
